# Ecological drivers of global gradients in avian dispersal inferred from wing morphology

**DOI:** 10.1038/s41467-020-16313-6

**Published:** 2020-05-18

**Authors:** Catherine Sheard, Montague H. C. Neate-Clegg, Nico Alioravainen, Samuel E. I. Jones, Claire Vincent, Hannah E. A. MacGregor, Tom P. Bregman, Santiago Claramunt, Joseph A. Tobias

**Affiliations:** 1 School of Earth Sciences, University of Bristol, Bristol, BS8 1TQ UK; 2 Department of Zoology, University of Oxford, South Parks Road, Oxford, OX1 3PS UK; 3 School of Biological Sciences, University of Utah, Salt Lake City, UT 84112 USA; 4 Department of Environmental and Biological Sciences, University of Eastern Finland, FI-80100 Joensuu, Finland; 5 School of Biological Sciences, Royal Holloway, University of London, Egham, Surrey, TW20 OEX UK; 6 Conservation Society of Sierra Leone, 86 Main Road, Freetown, Sierra Leone; 7 School of Biological Sciences, University of Bristol, Bristol, BS8 1TQ UK; 8 Future-Fit Foundation, 68 Hanbury St, Spitalfields, London, EC2A 2EX UK; 9 Department of Natural History, Royal Ontario Museum, Toronto, ON M5S 2C6 Canada; 10 Department of Ecology and Evolutionary Biology, University of Toronto, Toronto, ON M5S 1A1 Canada; 11 Department of Life Sciences, Imperial College London, Silwood Park, Ascot, SL5 7PY UK

**Keywords:** Macroecology, Zoology, Biogeography

## Abstract

An organism’s ability to disperse influences many fundamental processes, from speciation and geographical range expansion to community assembly. However, the patterns and underlying drivers of variation in dispersal across species remain unclear, partly because standardised estimates of dispersal ability are rarely available. Here we present a global dataset of avian hand-wing index (HWI), an estimate of wing shape widely adopted as a proxy for dispersal ability in birds. We show that HWI is correlated with geography and ecology across 10,338 (>99%) species, increasing at higher latitudes and with migration, and decreasing with territoriality. After controlling for these effects, the strongest predictor of HWI is temperature variability (seasonality), with secondary effects of diet and habitat type. Finally, we also show that HWI is a strong predictor of geographical range size. Our analyses reveal a prominent latitudinal gradient in HWI shaped by a combination of environmental and behavioural factors, and also provide a global index of avian dispersal ability for use in community ecology, macroecology, and macroevolution.

## Abstract (web summary)

In birds, the hand-wing index is a morphological trait that can be used as a proxy for flight efficiency. Here the authors examine variation of hand-wing index in over 10,000 bird species, finding that it is higher in migratory and non-territorial species, and lower in the tropics.

## Introduction

Dispersal plays a key role in ecological processes at a range of spatial and temporal scales^1,2^. The rate and distance of dispersal by organisms is thought to influence macroevolutionary patterns of speciation and extinction^3–5^, as well as macroecological patterns of geographical range^6–8^ and range overlap^9,10^, and is thus a major factor driving community assembly^11,12^. Within populations, dispersal is a critical component of meta-population and meta-community dynamics^13,14^, thereby regulating fundamental ecological processes including nutrient transfer, pollination and seed dispersal^15^. Variation in dispersal is also an integral factor in predicting biological invasions^16^, as well as species sensitivity to climate^17^ and land-use change^18,19^. Despite this broad relevance, however, we still know remarkably little about phylogenetic and geographical variation in dispersal and its underlying drivers^9,20^.

Dispersal distances vary widely in animals from small-scale movements in sedentary species to global journeys spanning both hemispheres in migratory species. On the one hand, this variation is thought to be largely driven by environmental factors. In particular, temporal variability in climate or resources is expected to favour increased mobility^1,2,20^, which in turn is expected to generate a latitudinal gradient in dispersal because of increased seasonality towards the poles (e.g. refs. ^18,21^). On the other hand, dispersal is expected to be linked to a range of species attributes only weakly correlated with latitude, including body size^22^, diet^23^ and resource defence strategy^24^. Estimating the relative roles of these environmental or ecological factors has proved challenging, however, because dispersal is difficult to quantify directly in most natural systems, particularly in a standardised way across large numbers of species^20,25,26^.

**Fig. 1:**
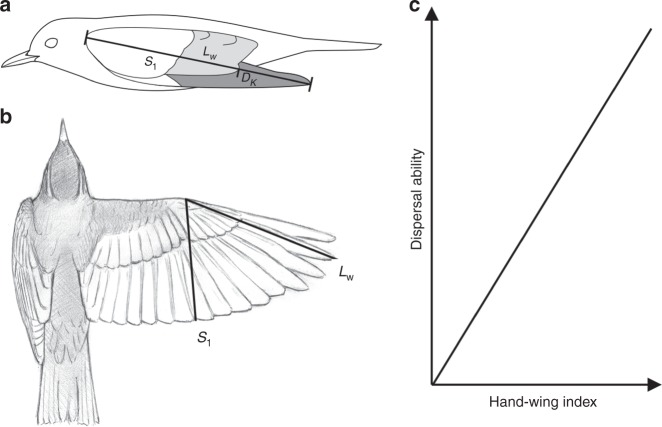
Calculating the hand-wing index (HWI). **a** Diagram showing linear measurements used to calculate HWI taken on a standard museum study skin (secondary feathers shown in pale grey; primary feathers in dark grey). Wing length (*L*_w_) is the distance from carpal joint to the tip of the longest primary feather; secondary length (*S*_1_) is the distance from carpal joint to the tip of the first secondary feather; Kipp’s distance (*D*_K_) is the difference between *L*_w_ and *S*_1_. **b** Open wing of a passerine bird showing how *L*_w_ and *S*_1_ are related to the wing’s span and width, and hence to its aspect ratio. **c** Because it is correlated with the aspect ratio, HWI is in theory positively associated with flight efficiency and key aspects of dispersal ability, including dispersal distance and gap-crossing ability.

Standard methods for quantifying dispersal, such as mark-recapture, GPS tracking and estimates of gene flow, are time-consuming, expensive, and difficult to scale. Even in vertebrates, comparative studies of dispersal have been limited to very small sample sizes, typically in well-studied organisms and regions. For example, natal and breeding dispersal data were made available for 75 British bird species based on nearly 100 years of intensive mark-recapture data^22^, while a recent survey of mammalian movement was based on GPS data from only 57 species^27^. Until such measurements become easier to implement at a wide scale, the most promising approach for comparative analyses relies on standardised biometric indices of dispersal. Perhaps the most familiar of these indices, the hand-wing index (HWI), is a morphological metric linked to wing aspect ratio^28,29^ and widely used as a single-parameter proxy of avian flight efficiency and dispersal ability^10,30–36^. HWI has become a mainstay of macroecological analyses partly because – unlike direct measurements of wing-aspect ratio, which requires measurement of wing area on open wings – it can be calculated from measurements obtained from dried museum specimens (Fig. 1a, b). Current species sampling for HWI, however, remains taxonomically incomplete and biased towards temperate regions.

To provide a global synthesis of variation in avian HWI, we directly measured the wing morphology of 41,981 museum specimens and live birds representing 10,338 (>99%) bird species. For each species, we calculated average HWI from a combination of linear wing measurements, then mapped phylogenetic and spatial variation in HWI to investigate the global inter-specific drivers of avian wing morphology. Birds – the largest tetrapod radiation – provide an ideal test case for a broad-scale analysis of dispersal morphology because they are globally distributed and reasonably well studied, with a full species-level phylogeny^37^ and complementary datasets on geographical distribution, along with a range of ecological, behavioural and other life history variables^38–40^.

We first use spatial mapping to visualise the geographic distribution of HWI and then apply Bayesian phylogenetic mixed models to explore the mechanisms underlying this pattern. We include two biogeographic, four climatic, and five ecological variables to assess which of these factors best explain interspecific variation in HWI, both across all birds and separately within major groups (passerines versus non-passerines). We also test the hypothesis that wing morphology drives variation in geographical range size and use separate models to further explore the link between HWI and migration. Given that a growing body of evidence suggests that HWI predicts flight efficiency and dispersal ability in birds (Fig. 1c)^28,35,41^, our results provide insight into the factors shaping the evolution of dispersal-related traits across larger spatial and temporal scales, as well as the consequences of such traits for widely observed biogeographic patterns.

## Results

### Patterns of variation in HWI

**Fig. 2:**
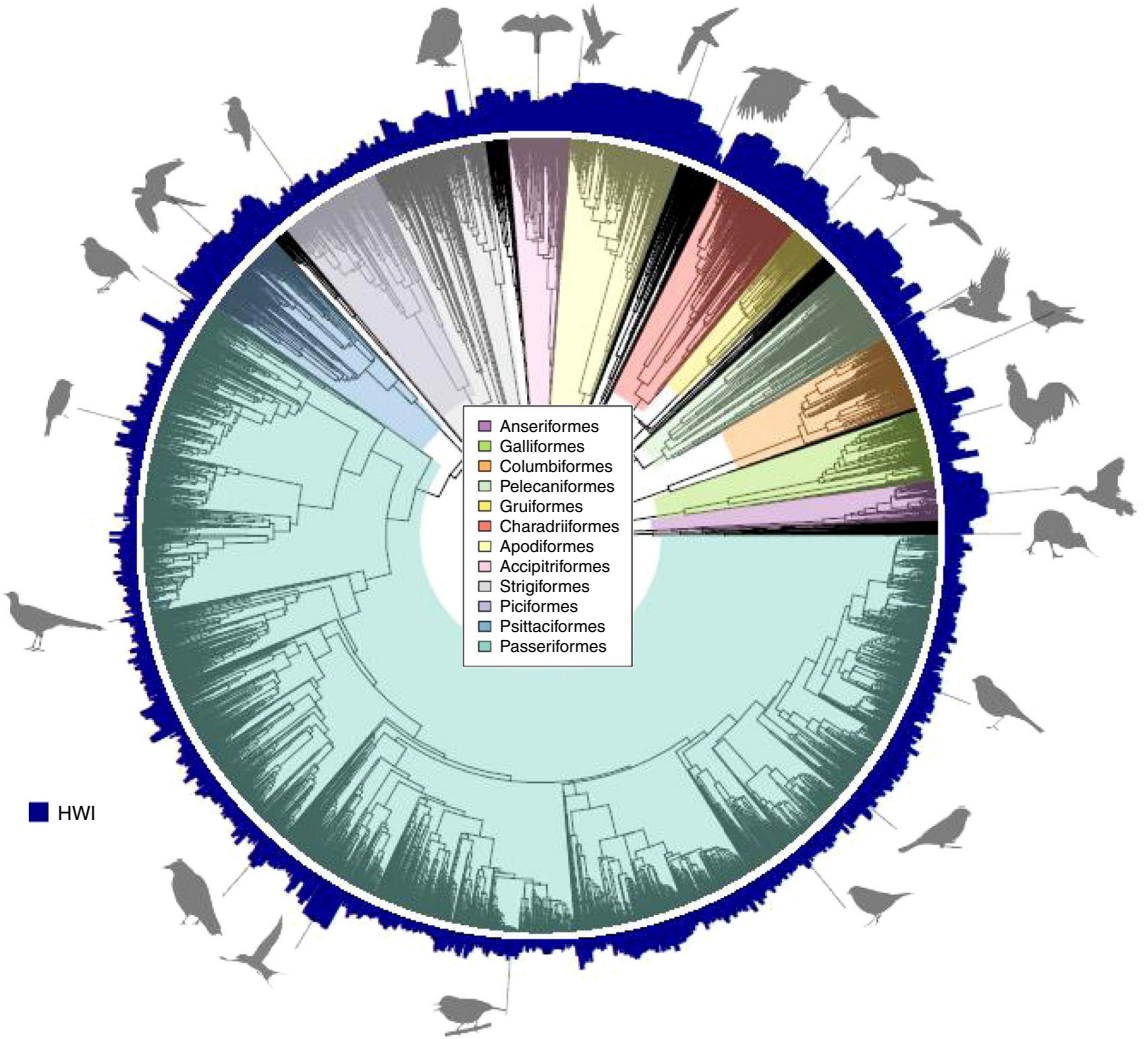
The phylogenetic distribution of hand-wing index (HWI) values across birds (*n* = 9945 species). Variation in HWI is plotted at branch tips of a single phylogenetic tree extracted from www.birdtree.org using the Hackett backbone^37^. For ease of interpretation, the 12 most species-rich clades are highlighted. Silhouettes are from phylopic.org, unchanged but for grey-scaling, with credit to Alexandre Vong, Andrew Butko, Arthur Grosset, Avenue, Aviceda, Bennet McComish, Brant C. Faircloth, Dori, Emily Willoughby, HuttyMcphoo, John E. McCormack, L. Shyamal, Lip Kee Yap, Mark Hannaford, Michael G. Harvey, Nevit Dilmen, Nicolas G. Crawford, Prin Pattawaro, Rebecca Groom, Robb T. Brumfield, T. Michael Keesey, Travis C. Glenn. The CC BY-SA 3.0 license can be found at https://creativecommons.org/licenses/by-sa/3.0/ and the CC BY 3.0 license at https://creativecommons.org/licenses/by/3.0/. Information on CC0 1.0 (no copyright) can be found at https://creativecommons.org/publicdomain/zero/1.0/. Further image information is available in the Supplementary Notes.

Across all birds, HWI ranges from 0.016 in *Rhea pennata* to 74.8 in *Phaethornis ruber*. At the scale of orders, HWI is lowest within the ratites (e.g. Struthioniformes, mean 0.019) and highest for the tropicbirds (Phaethontiformes, mean 69.2) (Fig. 2). Viewing total phylogenetic variation in this trait reveals that, on average, HWI values are typically lower in passerines than non-passerines (Fig. 2). Notable peaks in HWI coincide with highly dispersive clades such as parrots (Psittacidae), pigeons (Columbidae), shorebirds (Charadriiformes), seabirds, and waterfowl, as well as groups specialised on foraging in flight, such as swallows (Hirundinidae) or swifts and hummingbirds (Apodiformes).

**Fig. 3:**
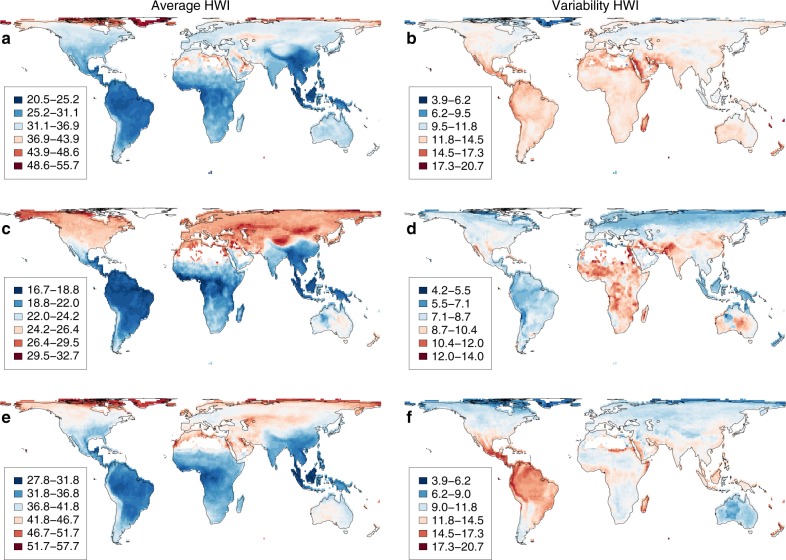
Global variation in hand-wing index (HWI). Maps are shown for all birds (**a**, **b**; *n* = 8504), passerines (**c**, **d**; *n* = 5153), and non-passerines (**e**, **f**; *n* = 3351). HWI is calculated for grid-cell assemblages as both average (**a**, **c**, **e**) and variability (**b**, **d**, **f**). Each grid cell represents a 1° × 1° square (~110 km × 110 km).

Focusing on geographical variation (Fig. 3), we find that the highest average HWIs are found in scattered regions worldwide, notably in the high Arctic, and in drylands such as the Saharan and Arabian deserts. In addition, a pronounced latitudinal gradient in HWI is clearly visible, with the lowest values consistently found in tropical regions. These spatial patterns are largely recapitulated in both non-passerines and passerines, although the latitudinal gradient is shallower in passerines with relatively high HWI much more broadly distributed across the temperate zone, particularly in the northern hemisphere. This apparent relationship between HWI and certain biomes suggests that avian flight ability is broadly related to climatic conditions, in particular environmental variability (e.g. seasonality).

Focusing on trait variability within assemblages (Fig. 3), we find that the highest variability in HWI is in the Saharan and Arabian deserts, the Andes mountains, Madagascar, and the Pacific islands (e.g. New Zealand, New Caledonia, Fiji, Hawaii, and the Galapagos). In other words, these are hotspots of variability supporting a wide spectrum of dispersal traits from low to high HWI. Different patterns emerge within major clades: variability in passerine HWI peaks in Africa, Australia, and south-central Asia from Iran to Pakistan, whereas in non-passerines variability peaks in New World low latitudes. Thus, the co-occurrence of different HWI values is driven not only by environment, but by the separate evolutionary and biogeographic histories of different clades.

### Ecological and environmental drivers of HWI

**Fig. 4:**
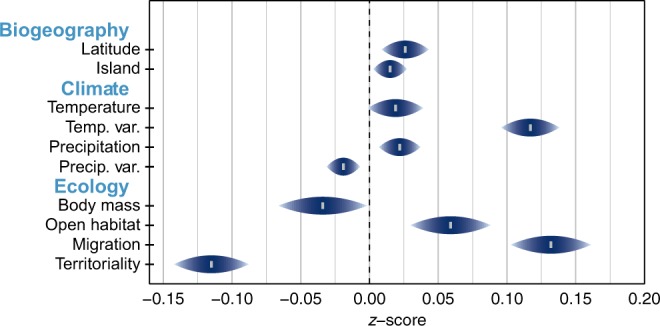
Predictors of hand-wing index (HWI) in birds (*n* = 9273 species). Shown are *z*-scores and 95% credible intervals (CI) computed with Bayesian phylogenetic mixed models; the dashed line represents a coefficient of 0. High *z*-score indicates positive association with HWI; low *z*-score indicates negative association with HWI. Climatic variables are calculated for each 1° × 1° grid cell of geographical range and averaged. Temperature annual mean temperature, Temp. Var. variation in monthly temperature values over a year (standard deviation), Precipitation annual precipitation, Precip. Var. variation in monthly precipitation values over a year (coefficient of variance), Open Habitat grasslands, deserts, coasts, oceans. Dietary categories are omitted for ease of interpretation. See Supplementary Table 1 for more information.

**Fig. 5:**
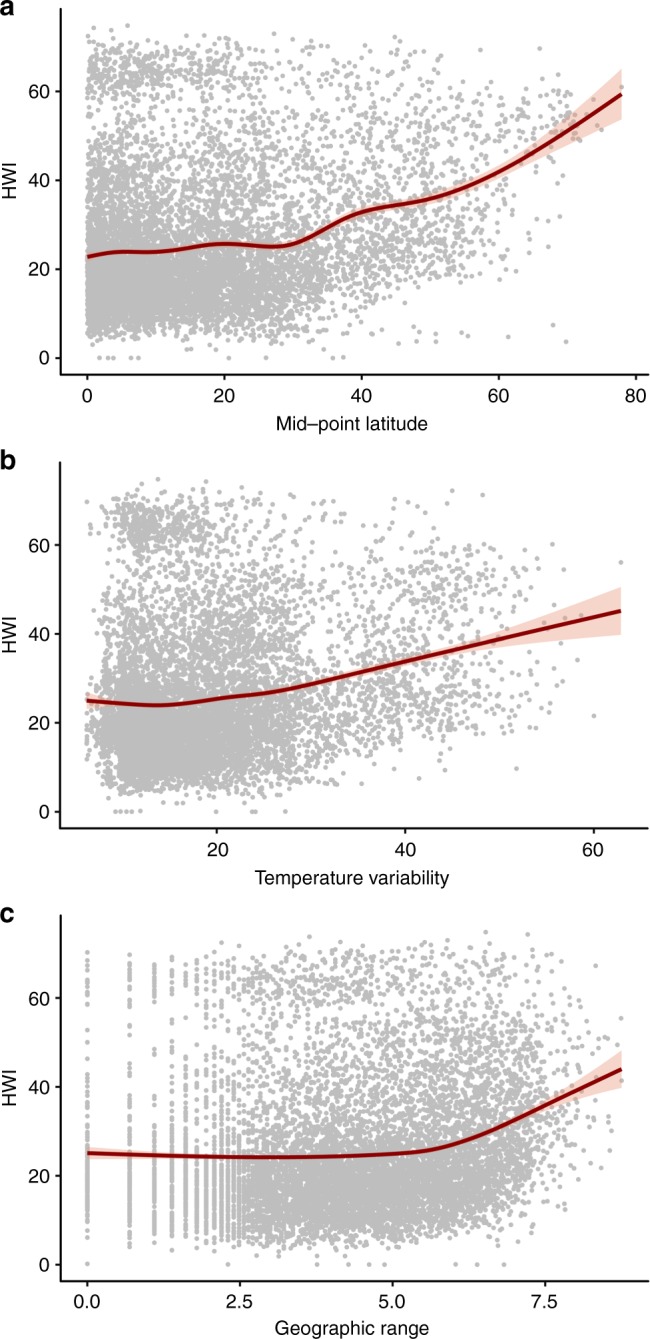
Relationship between hand-wing index (HWI) and environmental variables. Panels show how HWI of all birds (*n* = 9353 species) varies with (**a**) latitude, (**b**) temperature variability, and (**c**) geographical range size. All environmental variables are generated from polygons of breeding ranges; climatic variables are calculated for each grid cell of the range and averaged; overall range sizes (km^2^) are log-transformed. Red line shows model fit and pink shading shows 95% confidence intervals.

**Fig. 6:**
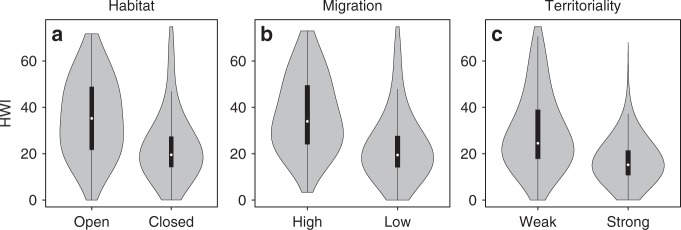
Relationship between hand-wing index (HWI) and ecological variables. A global sample of bird species (*n* = 9849) were classified according to (**a**) primary habitat (open grassland, deserts, shrubland, parkland, thorn forest, seashores, cities, closed forests), (**b**) migration (High >50% geographical range entirely vacated during non-breeding season, Low sedentary, elevational migrants, partially migratory, or <50% geographical range migratory), and (**c**) territoriality (weak defending territories only seasonally or never holding territories except very small lek or nest-site territories, strong defending territories year-round). Black interior boxplots show median (white dot) and first and third quartile (ends of black box). Whiskers (vertical black lines) indicate data minimum and maximum excluding outliers (calculated as first and third quartile ±1.5 times the inter-quartile range).

As potential predictors of variation in dispersal, we quantified a range of factors for each species, including latitude, climate, association with islands, migration, habitat, diet and territorial behaviour (see Methods). Across all birds, the strongest predictors of HWI are migration and breeding range temperature variability (*z* = 0.132 and *z* = 0.117, respectively; see Figs. 4–6, Supplementary Table 1), two factors that are themselves correlated because migration tends to arise in species breeding in highly seasonal environments (see Tables S7–9). We also found that HWI is strongly negatively correlated with year-round territory defence (*z* = −0.115, Fig. 6), presumably because this behaviour is tightly bound to a relatively sedentary lifestyle. We find further significant, though weaker, correlations with diet, habitat type and association with islands, as well as latitude and climate. Specifically, high HWI is associated with nectarivory, open habitats, high precipitation, high temperatures, high latitudes and greater association with islands. Conversely, omnivores, invertivores or species that breed in locations with high precipitation variability are more likely to have low HWI. Although body mass is traditionally used as an index for dispersal in vertebrates, with larger size assumed to indicate greater dispersal ability (e.g. refs. ^42,43^), we find a slight and non-significant negative correlation between avian body mass and HWI (*z* = −0.034, *pMCMC* = 0.060). This result is not surprising given that some of the largest bird species (e.g. ratites including ostriches, rheas and cassowaries) are flightless with low HWI.

Across passerines, the correlates of HWI are similar to those for all birds, although temperature is no longer a significant predictor (see Supplementary Table 2). In non-passerines, HWI is correlated with neither diet nor breeding range precipitation seasonality nor association with islands (see Supplementary Table 3), but is weakly negatively correlated with body mass. The tendency within non-passerines for smaller species to have larger HWIs is perhaps strongly driven by swifts and hummingbirds (Apodiformes) being adapted for sustained flight whereas the largest non-passerines are flightless. The strong effect of migration on HWI is retained in all analyses and is not sensitive to different classifications of migration (Supplementary Tables 10–12).

### HWI as a predictor of biogeography and migration

When we examined the biogeographic consequences of wing morphology, we found that multiple variables are correlated with range size in birds, namely temperature variability, precipitation, diet, latitude, migration, association with islands and whether the species is found in the northern or southern hemisphere; there was also a weaker but still significant effect of habitat type and an interaction between latitude and hemisphere (Supplementary Table 4). Some of these relationships reflect the well-established tendency for range size to increase with latitude (Rapoport’s rule) in correlation with latitudinal gradients in several predictors of range size included in our multivariate model (e.g. temperature variability, migration and diet). Additional interactions with hemisphere are probably related to asymmetries in land-area, with a much larger extent of available land-area in the northern hemisphere accommodating larger range sizes. After accounting for all these ecological and geographic factors, we found that species with higher HWIs have larger range sizes (*z* = 0.126). The findings are qualitatively similar when the model is restricted to non-passerines or passerines (Supplementary Tables 5 and 6) and re-run using different sources of migration data (Supplementary Tables 13–15).

HWI is the strongest predictor of migration across all birds (*z* = 2.279; Supplementary Table 7), as well as in non-passerines and passerines, separately. Additional analyses show that this result is consistent regardless of the source of migration data (Supplementary Tables 16–18). The only other predictors strongly and positively associated with migration across all taxonomic categories are temperature variability and latitude (Supplementary Table 7). In contrast with range size models, there are differences in secondary predictors of migration between non-passerines and passerines, including factors such as diet, climate and hemisphere (Supplementary Tables 8 and 9). These findings confirm that HWI predicts migration, but it is difficult to infer underlying mechanisms because wing morphology could be the cause or consequence of long-distance movement – indeed, our results suggest that a combination of these effects explains relatively tight co-evolution between HWI and migration reported in previous studies^25,28,33,44^.

## Discussion

Our global analysis of avian wing morphology reveals strong gradients in HWI shaped by environmental and ecological variables. We find that increased HWI is linked to both migration and reduced territoriality, as well as high variability in breeding-range temperature, with habitat type and diet as secondary effects. The most obvious spatial pattern is a latitudinal gradient in HWI from high values in polar regions to low values at the equator, suggesting that average dispersal ability declines in bird species that live in tropical regions. Likely contributing to this finding, tropical species often have sedentary lifestyles or year-round territoriality, both of which are promoted by low variability in breeding range temperature^39^. These broad-scale variations in dispersal ability may contribute to a number of pervasive macroecological and macroevolutionary patterns, including latitudinal gradients in diversification rate^45^, species richness^46^ and range size^47^, as well as the heightened sensitivity to environmental change reported for tropical taxa^18,48,49^.

Previous studies mapping inter-specific variation in dispersal indices have shown few consistent geographical trends. This may reflect poor data quality and coverage, because well-sampled indices are limited to indirect factors such as body size, whereas more directly informative measurements are costly and time consuming to measure, with coverage only available for relatively small subsets of species or regions (e.g. refs. ^27,33,50,51^). HWI offers a useful compromise because it is both amenable to global sampling and mechanistically linked to flight ability^28,35,41^. The extent to which HWI is associated with average dispersal distance for each species requires further empirical testing, but even sedentary tropical species with high HWI (e.g. parrots, pigeons and hummingbirds) are typically associated with increased barrier- or gap-crossing ability^19,52^, a critical factor shaping patterns of gene flow^32^, range expansion^9^ and responses to habitat fragmentation^18^. Our analyses thus provide unique insight into the broad-scale distribution of dispersal limitation and its effects, at least until direct dispersal estimates can be sampled with standardised methods across thousands more species.

Although HWI clearly increases with latitude, our models reveal that latitude itself is not the key factor driving differences in wing morphology. We find much stronger evidence that climatic variability – or seasonality – explains differences in dispersal ability. In particular, temperature variability is one of the strongest predictors of HWI, with an effect nearly five times that of latitude. Temperature variability appears to be fundamental because its effect is far (~5–6×) greater than either temperature or precipitation, both of which are relatively weakly correlated with wing morphology. Conversely, in passerines, variability in precipitation has a negative relationship with HWI, perhaps because sedentary species abound in tropical regions with comparatively stable temperature regimes but pronounced wet seasons.

A link between temporal climatic variability and increased dispersal has been demonstrated in a variety of taxa^2,53^, in line with theoretical predictions^20,54^. Overall, our findings support the prevailing view that species inhabiting stable environments are more likely to be sedentary, thus lacking dispersal-related adaptations, whereas other species inhabiting uncertain or highly seasonal environments can thrive in these conditions by relocating in space, often over long distances.

The connections between HWI, climate and latitude appear to be mediated by ecological and behavioural traits. In particular, territoriality and migration are strongly related to both latitude and seasonality, with year-round territory defence widespread in tropical species, and migration far more prevalent towards the poles. Year-round territoriality is a resource-defence strategy associated with reduced HWI in our analyses, whereas migration is linked to increased HWI. Since year-round territoriality has an effect similar in strength and direction to that of low migration in a multivariate model, our results suggest that these effects are not simply correlations. Rather, in both migratory and non-migratory species, those that hold year-round territories have lower HWI than those that do not. These findings are consistent with ecological theory predicting that individuals or species defending resources will have lower rates of dispersal compared with less territorial individuals or species^1,2,53^.

Diet is another factor partially associated with latitude or environmental variability, although the underlying relationships with HWI are complex. For example, previous studies have suggested that insectivory is associated with larger dispersal distances than other trophic niches^23^, reflecting the traditional focus on temperate-zone systems where insectivorous birds tend to be migratory in response to seasonal prey availability. In contrast, tropical insectivores are mostly sedentary, and often year-round territorial^21^, which explains why low-latitude insectivores have lower HWI than most other dietary niches in tropical systems. Nectarivores, by contrast, have the highest average HWI whether viewed across all birds or only in passerines. Moreover, nectarivore HWI does not tend to decline towards the tropics, presumably because of behavioural aspects of the foraging niche^40^. Strong flight ability makes biological sense in this guild because many nectarivores either forage on the wing or move across large areas to exploit patchy and unpredictable resources^55^. Specialist nectarivores are largely confined to the tropics, yet this reverse trend of high HWI in a tropical guild is not detected at global scales because nectarivores are greatly outnumbered by members of other trophic niches (e.g. insectivores, granivores and omnivores) that have relatively low HWI in the tropics.

According to speciation theory, dispersal is a key factor determining the likelihood of speciation in a given geographical area because it influences rates of gene flow across barriers^5,56^. On islands, speciation is most likely to occur when dispersal is high enough to promote island colonisation^8,33^ but not so high that gene flow is too frequent^30,57^. On continents, however, most evidence suggests that low dispersal promotes avian diversification (e.g. refs. ^32,35,56,58^). Even on larger islands, such as Borneo, low dispersal (inferred from low HWI) strongly predicts reduced gene flow and incipient speciation in birds^34^. Thus, our finding of a latitudinal gradient in HWI is consistent with the view that dispersal constraints promote allopatric speciation in tropical birds, contributing to the latitudinal diversity gradient^21^.

Dispersal has also been linked to geographical range size and range overlap. For example, it is often assumed that strong dispersal facilitates colonisation of new regions, thereby driving range expansion, although empirical evidence linking dispersal and range size is largely inconclusive^6,7,26^. Our global analyses show a positive, though weak, correlation between avian HWI and range size, matching the findings of previous studies focused in different taxonomic groups^59^ or at smaller taxonomic scales^33^. Some studies also find evidence that variation in avian HWI predicts species coexistence^9^, with both range expansion^36^ and the initial establishment of range overlap^10^ occurring more readily among lineages with higher HWI. If avian dispersal increases with latitude, as suggested by spatial patterns in HWI, then this could help to explain why the same trend from tropics to poles arises in both range size (Rapoport’s rule)^47^ and the incidence of early range overlap (i.e. sympatry)^60^.

Finally, global variation in HWI may highlight species and communities at risk from climate or land-use change. For example, our findings support previous suggestions that gap- or barrier-crossing ability declines towards the equator^18,52^ and that many tropical birds are therefore more susceptible to habitat fragmentation^18,48^. HWI can also be used to study the role of dispersal ability in determining species responses to habitat fragmentation and configuration^61^. In addition, given the importance of dispersal as a key factor influencing how individual organisms and populations respond to environmental change, species-level variation in HWI may serve as a standardised trait for modelling historical biogeographic patterns (e.g. Sukumaran et al.^62^) as well as the effects of future climate and development scenarios on biodiversity^17,27^. Ongoing changes in landscape and climate are leading to increased distances between habitat patches, seasonal ranges, and migration stopovers^63^. Ultimately, these future landscapes are predicted to select for greater dispersal ability, suggesting that adaptation may even cause gradients in HWI to shift or steepen over time.

We have shown that three inter-connected factors – migration, year-round territoriality, and environmental variability – are most strongly correlated with variation in HWI, a standard index of dispersal ability in birds^10,31,35^. These findings reveal that environmental and behavioural factors act in tandem to shape biogeographic gradients in dispersal traits and suggest a prominent pattern of reduced dispersal ability in tropical bird species^21,48^. This latitudinal gradient in dispersal traits has received little attention, but we suspect it has far-reaching implications. In particular, our analyses highlight the potential roles of dispersal ability in driving more familiar macroecological patterns, including latitudinal gradients of speciation, range size and sensitivity to environmental change, while also providing a trait-based template for exploring these roles at a range of scales from local communities to global ecosystems.

## Methods

### Avian hand-wing index

The hand-wing index (HWI) is here defined as 100*D_K_/*L*_w_, where *D*_K_ is Kipp’s distance (the distance between the tip of the first secondary feather and the tip of the longest primary feather) and *L*_w_ is wing length (see Fig. 1). In effect, HWI is Kipp’s distance corrected for wing size. A swallow (Hirundinidae) and a partridge (Phasianidae) might have similar Kipp’s distance but highly divergent HWI, with the latter providing a better index of differences in their flight efficiency. High HWI indicates elongated (high aspect ratio) wings suitable for efficient long-distance flight, whereas low HWI indicates broader (low aspect ratio) wings associated with weaker or short-distance flight^31,35,41^. While these morphological differences provide a clear mechanistic link between HWI and flight efficiency or dispersal potential, the connection with actual dispersal is theoretically weaker because some species may be highly adapted for airborne lifestyles yet relatively sedentary. Nonetheless, both Kipp’s distance and HWI have been found to relate to inter-specific variation in dispersal distances and migratory behaviour^25,28,35,41,64^. Flightless birds have the lowest HWI^31^.

We compiled Kipp’s distance (*D*_k_) and the length of the unflattened wing chord (*L*_w_) for 45,801 museum specimens and live birds representing 10,338 extant and recently extinct species (see Supplementary Methods). To allow global-scale phylogenetic analyses, we assigned species limits according to the Jetz et al.^37^ global bird phylogeny, adopting recent taxonomic revisions or newly described species where possible. We selected at least two males and two females in good condition for measurement when available, giving a mean of 4.43 individuals measured per species. We restricted our sample to adults, excluding all specimens labelled or identified in-hand as juveniles. Measurements from museum specimens were taken from the nominate subspecies whenever possible. We accessed specimens of five kiwi species (order Apterygiformes) but were unable to measure Kipp’s distance as the wings are vestigial (lacking distinct primary and secondary feathers). We therefore excluded all kiwi species from our analyses, but for completeness we include them in our phylogram (Fig. 2), global map (Fig. 3) and dataset following the convention that kiwi HWI = 0.1, in line with other flightless ratites^31,65^.

The majority (68%) of measurements were taken by 8 observers (the authors), but a further 84 observers contributed to the dataset by providing measurements from specimens accessed at a total of 73 collections and field sites (see Supplementary Notes). To maximise consistency, all contributors were supplied with a detailed protocol for taking wing measurements. To assess the effect of observer biases, we collected 220 replicate wing measurements by different measurers for 146 species (see Supplementary Methods). Measurer identity explained ~0.5% of the variation in Kipp’s distance and less than 0.01% of the variation in wing chord (Supplementary Fig. 1). These findings support previous analyses concluding that independent biometric trait measurements by different observers are very highly correlated and thus unlikely to influence multi-species analyses at macroecological scales^41,65,66^.

Pooling measurements from live birds and preserved museum specimens is potentially problematic given post-mortem shrinkage of wing feathers^67^. When we compared museum- and field-based samples within species (*n* = 362), however, the measurement type explained <0.002% of variation in wing chord and 0.35% of variation in Kipp’s distance (Supplementary Fig. 2). Post-mortem feather shrinkage therefore appears to be negligible and unlikely to explain macroecological patterns at the scale addressed in this study.

### Ecological and behavioural variables

We assigned all species to dietary guilds based on the classification of Pigot et al.^66^, which was adapted from published diet scores^38^ and updated with information from primary and secondary literature. We merged all four vertivore categories (vertebrate-eaters, including carnivores and piscivores) into a single grouping. Any species with >50% of its diet belonging to a single category, or with exactly 50% of its diet in one category and <50% in all other categories, was classified as belonging to that majority category as a guild; all other species were classified as omnivores.

Territorial behaviour was classified into a binary score based on data obtained from Tobias et al.^39^. Specifically, we treated species as strongly territorial if they defend year-round territories, and weakly territorial if they either lacked any form of territorial behaviour or only defend seasonal territories, including nest-sites and mating display sites. Habitat use was also classified into a binary score according to data in Tobias et al.^39^: closed habitats include forests and semi-open habitats such as parkland, shrubland and marsh vegetation; open habitats include grasslands, deserts, coasts and oceans. Migration data was obtained from BirdLife International^68^, with ‘full migrants’ scored here as migratory and all others (partial migrants, altitudinal migrants, non-migrants, and nomads) scored as non-migratory. To check whether our results were influenced by this scoring system, we re-ran analyses with migratory data obtained from two other global databases^39,69^ (see Supplementary Tables 10–18). Body mass data was published by Tobias & Pigot^40^, based largely on Dunning^70^ with updates from primary and secondary literature.

We computed geographical range size by intersecting global range polygons^68^ with a 1° × 1° grid and counting the number of grid cells overlapped by each polygon. These ranges were then intersected with data from WorldClim v. 1 to obtain average annual temperature, temperature variability, annual precipitation, and precipitation variability for all species^71^. As an estimate of ‘association with islands’, we combined the same range polygons for each species with a land GIS layer and quantified the proportion of ranges intersecting islands with landmass below 2000 sq. km^10^. We extracted median range latitude from range polygons and entered this into models as an absolute (unidirectional) value. For data display purposes, range maps were intersected with a 1° × 1° grid (~110 km × 110 km at the equator), and the traits of the species living within each grid cell were plotted using the R package sf^72^.

### Causes of variation in HWI

Bayesian phylogenetic generalised linear mixed models were run using the R package MCMCglmm^73^. We tested the effect of multiple variables – body mass, migration, diet, territoriality, latitude, association with islands, and climatic factors – on mean species HWI. We combined all variables as factors in a single model, with phylogeny as a random effect. Correlations between factors were minor or moderate, with the exception of stronger correlations among climatic factors associated with latitude (Supplementary Table 19). All quantitative variables were scaled to have a mean of 0 and a variance of 1; range size and HWI were log-transformed; and the association with islands variable was arcsine-transformed. Priors were initially set using inverse-Wishart priors for the phylogenetic and residual variance (*V* = 1, *ν* = 0.002) and diffuse normal priors for the fixed effects (mean 0, variance 10^10^). After conducting a dummy run of 11,000 iterations on an arbitrary tree with a burn-in of 1,000 and a thin of 50 to determine a start point for the R- and G-structures, each of 100 tree topologies was run sequentially for 15,000 iterations with a burn-in of 5,000 and a thin of 1,000, for a total posterior sample of 1,000 solutions (10 per tree). All chains were visually inspected to ensure proper mixing, and autocorrelation was checked using the command ‘autocorr’ with 0.1 used as a target threshold. Finally, when we calculated the variance inflation factors (VIF) of variables in the main model, we found that all were lower than 6 (Supplementary Table 20). This is well below the suggested threshold of 10, suggesting that correlation among variables is low and that parameters can be interpreted individually^74^. We therefore retained all predictor variables (see Supplementary Methods).

### Consequences of variation in HWI

Some factors are perhaps better viewed as consequences, rather than causes, of wing morphology. Two possible examples are geographical range size and migratory strategy. We therefore ran additional models testing the predictive effects of wing morphology on geographical range size to assess whether HWI influences the extent of the global distribution of bird species. As potential co-variates, we included migration, habitat, body mass, diet, association with islands, a latitude-hemisphere interaction and the same four environmental variables (temperature, temperature variability, precipitation and precipitation variability).

Previous studies showing a link between migration and HWI in birds have usually interpreted wing morphology as an adaptation to migration. This view is supported by the observation that HWI can be influenced by switches in migratory strategy. Adaptations for dispersal such as high HWI can be rapidly lost in sedentary or insular taxa^33,44,56^, suggesting that HWI can also readily evolve in migratory lineages. Nonetheless, an alternative view is that particular wing morphologies may promote migration, for example if sedentary species with higher HWI are more likely to evolve long-distance dispersal strategies than those with lower HWI. To examine this hypothesis, we tested whether HWI predicted migration rather than vice versa. As with the analyses testing for effects of HWI on geographical range size, we included the same set of potential co-variates (except migration). As migratory strategy is here considered a binary variable, we used a logistic regression (MCMCglmm family ‘categorical’) with priors for the fixed effects set using the command ‘gelman.prior’, an improper prior for the phylogenetic variance (*V* = 10^−10^, *ν* = −1), and the residual variance fixed at 1.

All phylogenetic analyses were conducted on a sample of 100 trees obtained from the Hackett backbone of the global bird phylogeny (www.birdtree.org)^37^. To assess how results varied across different avian clades, we ran models separately for all birds, passerines, and non-passerines.

### Reporting summary

Further information on research design is available in the Nature Research Reporting Summary linked to this article.

## Supplementary information

Supplementary Information

Reporting Summary

## Data Availability

The morphological and ecological data used in this study is available at 10.5281/zenodo.3747657. Range information and migration data is publicly available from www.birdlife.org; climate data from www.worldclim.org; phylogenetic data from www.birdtree.org; territoriality, habitat use, and additional migratory data from Tobias et al.^39^.

## Code availability

The code used to conduct the analyses and generate the figures is available on request from the authors.
