# Supplementary material for: Ecological drivers of global gradients in avian dispersal inferred from wing morphology
Source: Nat Commun. 2020 May 18;11:2463. doi: 10.1038/s41467-020-16313-6 (PMC7235233; doi:10.1038/s41467-020-16313-6)
Supplement: Supplementary file 1 — Supplementary Information [file 41467_2020_16313_MOESM1_ESM.pdf]

Ecological drivers of global gradients in avian dispersal inferred from wing morphology

Catherine Sheard, Montague H. C. Neate-Clegg, Nico Alioravainen, Samuel E. I. Jones, Claire Vincent, Hannah E. A. MacGregor, Tom P. Bregman, Santiago Claramunt & Joseph A. Tobias

SUPPLEMENTARY MATERIAL

Supplementary Methods

Supplementary Figures (1-2)

Supplementary Tables (1-20)

Supplementary Notes

Supplementary References

Supplementary Methods

Trait measurement

We measured Kipp's distance (D_k) directly except when the only measurements available were secondary length (S_1). In these cases, we calculated D_k by subtracting S_1 from wing length (L_w). All wing measurements were taken to the nearest 0.5 mm.

Evaluating data quality

Wing measurements were taken by multiple observers following a defined protocol designed to standardise data, but some degree of observer bias caused by differences in measurement technique is inevitable. To assess the extent of observer bias, we assembled a set of duplicate wing measurements made by 28 measurers on 220 museum specimens. This sample spanned 148 bird species from 17 orders and 65 families. We fitted linear mixed models with order, family, genus, and species as nested random effects using the R package lme4[1], and calculated R^2 values using the package MuMIn[2]. We found that measurements taken by different observers were highly correlated, and that interspecific differences explained 98% of the variation in Kipp's distance and over 99% of the variation in wing chord (see Supplementary Fig. 1). Measurer identity accounted for approximately 0.5% of the variation in Kipp's distance and less than 0.01% of the variation in wing chord, suggesting that observer bias does not affect the results of our macroecological analyses.

Another potential source of bias in our dataset is created by use of measurements taken on live birds versus preserved museum skins, due to minor post-mortem feather shrinkage in museum skins. Given that the vast majority of our measurements were based on museum specimens (43,013 of 45,801, 94%), this bias would be unlikely to have a large effect. To assess the accordance of measurements taken in each context, however, we compared measurements in the subset of species for which we had both field and museum measurements (362 species across 10 orders and 65 families, represented by a total of 4,018 specimens). As with evaluating observer bias, we fitted linear mixed models with order, family, genus, and species as nested random effects using the R package lme4[1], and calculated R^2 values using the package MuMIn[2]. Again, field- and museum-based measurements were highly correlated and differences between them negligible at macroecological scales, accounting for less than 0.002% of the variation in wing chord measurements and less than 0.35% of the variation in Kipp's distance (see Supplementary Fig. 2).

The use of species mean values to represent variation of morphological traits may be inappropriate if traits vary widely within species. However, this problem is much reduced at large taxonomic scales. Previous global analyses on avian morphological traits, including wing measurements, indicate that the vast majority (>98%) of trait variance can be assigned to inter-, rather than intra-, specific variation, indicating that the use of average trait values is appropriate at this scale[3].

Alternate measures of migration

Analyses using migration data may be sensitive to the scoring system used to classify species. To assess whether this may influence our results, we obtained additional migration scores from Tobias, et al. [4] and Eyres, et al. [5]. For the former source, 'migratory' species were here classified as migratory, while 'sedentary' and 'partially migratory' species were here classified as non-migratory. For the later source, 'directional migratory' species were here classified as migratory, while 'dispersive migratory', 'nomadic', and 'resident' species were classified as non-migratory. Despite some mismatch in coding schemes, the differences in results are all minor (Supplementary Tables 10-18).

Multicollinearity

The predictor variables included in our models are not entirely independent. To assess whether non-independence would be likely to affect our results, we ran pairwise correlations among all variables presented in the main model evaluating predictors of HWI across all birds (Supplementary Table 19). For ease of interpretation, these correlations are presented without phylogenetic correction. When comparing the correlation between two continuous variables, or a categorical and a continuous variable, we present the adjusted R^2 from a linear regression. For the six combinations of variables which involves testing whether a categorical variable is correlated with a binary variable, we calculate the null and residual deviance using a logistic regression and estimate a pseudo-R^2 as 1 – (residual deviance)/(null deviance).

We found that most (32) of the 55 pairwise comparisons have less than a 10% correlation. At the opposite extreme, four combinations of variables have a correlation above 50%: latitude and temperature, latitude and temperature variability, temperature and temperature variability, and precipitation and temperature variability. These correlations are to be expected inasmuch as they reflect latitudinal gradients in climatic conditions.

Although these raw comparisons reveal that few of our variables are affected by collinearity, the correlations on their own are not necessarily informative about the statistical validity of retaining each of these variables in our model. To explore this issue, we calculated variance inflation factors (VIFs), which provide a measure of the multicollinearity represented by each variable; that is, the extent to which a coefficient estimate may be influenced by correlations between multiple predictor variables. As a general rule, a VIF < 10 is considered low correlation.

VIFs were computed using a customised function, originally written by Austin Frank:

```
vif.MCMCglmm <- function (fit, intercept.columns = c(1)) {
  nF <- fit$Fixed$nfl
  v <- cov(as.matrix(fit$X[,1:nF]))
  nam <- colnames(fit$Sol[,1:nF])

  v <- v[-intercept.columns, -intercept.columns, drop = FALSE]
  nam <- nam[-intercept.columns]

  d <- diag(v)^0.5
  v <- diag(solve(v/(d %o% d)))
  names(v) <- nam
  v
}
```

In the main model (Fig. 4, Supplementary Table 1), the VIFs range from 1.07 (for scavenging diet) to 5.94 (for temperature variability), indicating a low effect of multicollinearity on the models presented. Again, this is to be expected because the environmental factors of primary interest – latitude, temperature, precipitation, seasonality – are imperfectly correlated. Arid regions, highly seasonal climates, and cold uplands span a range of latitudes, including the tropics and subtropics. A full list of VIFs is provided in Supplementary Table 20.

Supplementary Figures

Supplementary Figure 1. Correlations between duplicate wing measurements from different observers. Each of 220 specimens from 146 species were measured by two different people (n = 28 measurers). Points falling along the 1:1 line (shown in red) indicate a perfect correspondence between observers. R^2 values indicate the proportion of variation in observer 2's measurements explained by observer 1's measurements.

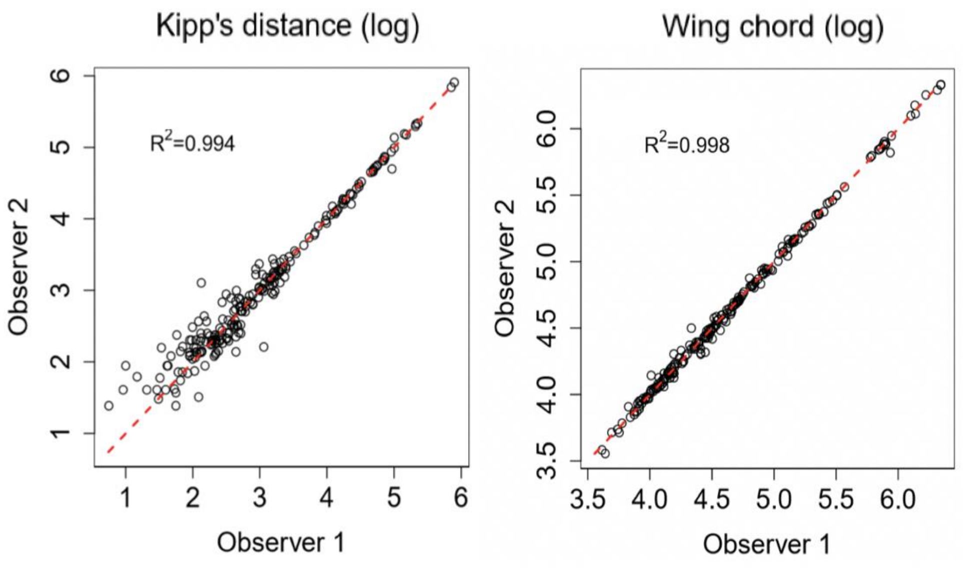

Supplementary Figure 2. Correlations between measurements taken from museum specimens and live individuals. Shown are average measurements from both sources, for the 362 species representing 10 orders and 65 families (n = 4,018 individuals). Points falling along the 1:1 line (shown in red) indicate a perfect correspondence between types of measurements. R^2 values indicate the proportion of interspecific variation in museum measurements explained by field measurements.

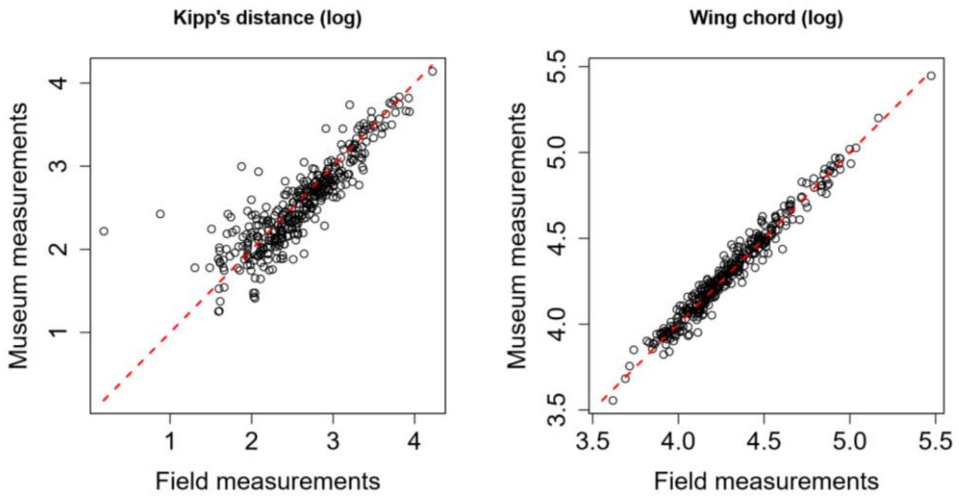

Supplementary Tables

Supplementary Table 1. Predictors of HWI for all birds calculated with a Bayesian phylogenetic mixed model (MCMCglmm). Variables with *p*-value estimates less than 0.05 are highlighted in grey. pMCMC represents double the probability that the coefficient estimate is above or below zero (whichever is smaller), based on the posterior distribution; CI = credible interval. Temperature = annual mean temperature. Temp. Var. = variation in monthly temperature values over a year (standard deviation). Precipitation = annual precipitation. Precip. Var. = variation in monthly precipitation values over a year (coefficient of variance). Lat. = median breeding range latitude (absolute value). See Figure 4 (main text) for visualisation. Model includes migration data from BirdLife International; kiwis (order Apterygiformes) have been omitted.

**Fixed effects**	*z*-score	lower 95% CI	upper 95% CI	pMCMC
Mass	-0.034	-0.068	-0.002	0.060
Island	0.015	0.005	0.027	0.008
Latitude	0.026	0.006	0.043	0.006
Temperature	0.019	0.001	0.039	0.048
Temp. Var.	0.117	0.095	0.138	<0.001
Precipitation	0.022	0.009	0.037	0.002
Precip. Var.	-0.019	-0.029	-0.007	<0.001
Territoriality	-0.115	-0.142	-0.088	<0.001
Diet -- invertebrates	-0.055	-0.098	-0.011	0.004
Diet -- nectar	0.089	0.010	0.187	0.048
Diet -- omnivores	-0.039	-0.077	0.002	0.046
Diet -- plants	-0.029	-0.110	0.049	0.480
Diet -- scavenger	0.046	-0.159	0.227	0.656
Diet -- seeds	-0.033	-0.081	0.026	0.244
Diet -- vertebrates	-0.065	-0.139	0.013	0.094
Migration	0.132	0.107	0.161	<0.001
Habitat -- open	0.059	0.028	0.088	<0.001
**Random effects**				
Phylogeny	0.707	0.584	0.847	
**Residuals**				
Residual	0.061	0.055	0.068	
**Summary statistics**				
DIC	4284.1			
R^2 estimate	0.964			

Supplementary Table 2. Predictors of HWI for passerines calculated with a Bayesian phylogenetic mixed model (MCMCglmm). Variables with *p*-value estimates less than 0.05 are highlighted in grey. pMCMC represents double the probability that the coefficient estimate is above or below zero (whichever is smaller), based on the posterior distribution; CI = credible interval. Temperature = annual mean temperature. Temp. Var. = variation in monthly temperature values over a year (standard deviation). Precipitation = annual precipitation. Precip. Var. = variation in monthly precipitation values over a year (coefficient of variance). Lat. = median breeding range latitude (absolute value). Model includes migration data from BirdLife International; kiwis (order Apterygiformes) have been omitted.

**Fixed effects**	*z*-score	lower 95% CI	upper 95% CI	pMCMC
Mass	0.005	-0.030	0.040	0.790
Island	0.026	0.006	0.046	0.012
Latitude	0.039	0.007	0.071	0.018
Temperature	-0.003	-0.034	0.029	0.836
Temp. Var.	0.167	0.122	0.204	<0.001
Precipitation	0.077	0.048	0.100	<0.001
Precip. Var.	-0.035	-0.054	-0.013	0.002
Territoriality	-0.165	-0.210	-0.120	<0.001
Diet -- invertebrates	-0.091	-0.174	-0.017	0.028
Diet -- nectar	0.161	-0.008	0.325	0.056
Diet -- omnivores	-0.111	-0.194	-0.048	<0.001
Diet -- plants	-0.333	-0.587	-0.080	0.010
Diet -- scavenger	0.130	-0.575	0.751	0.738
Diet -- seeds	-0.117	-0.223	-0.025	0.022
Diet -- vertebrates	-0.265	-0.619	0.121	0.160
Migration	0.233	0.182	0.280	<0.001
Habitat -- open	0.060	0.012	0.108	0.010
**Random effects**				
Phylogeny	0.962	0.828	1.110	
**Residuals**				
Residual	0.121	0.110	0.133	
**Summary statistics**				
DIC	6423.6			
R^2 estimate	0.961			

Supplementary Table 3. Predictors of HWI for non-passerines calculated with a Bayesian phylogenetic mixed model (MCMCglmm). Variables with *p*-value estimates less than 0.05 are highlighted in grey. pMCMC represents double the probability that the coefficient estimate is above or below zero (whichever is smaller), based on the posterior distribution; CI = credible interval. Temperature = annual mean temperature. Temp. Var. = variation in monthly temperature values over a year (standard deviation). Precipitation = annual precipitation. Precip. Var. = variation in monthly precipitation values over a year (coefficient of variance). Lat. = median breeding range latitude (absolute value). Model includes migration data from BirdLife International; kiwis (order Apterygiformes) have been omitted.

**Fixed effects**	*z*-score	lower 95% CI	upper 95% CI	pMCMC
Mass	-0.080	-0.126	-0.035	<0.001
Island	0.013	-0.004	0.030	0.130
Latitude	0.031	0.001	0.057	0.026
Temperature	0.040	0.002	0.072	0.030
Temp. Var.	0.098	0.068	0.132	<0.001
Precipitation	-0.034	-0.058	-0.013	0.004
Precip. Var.	-0.001	-0.019	0.018	0.880
Territoriality	-0.107	-0.162	-0.056	<0.001
Diet – invertebrates	-0.051	-0.131	0.030	0.240
Diet -- nectar	0.038	-0.102	0.194	0.596
Diet -- omnivores	0.022	-0.043	0.084	0.506
Diet -- plants	0.051	-0.038	0.168	0.304
Diet -- scavenger	0.053	-0.151	0.270	0.652
Diet -- seeds	0.054	-0.032	0.136	0.242
Diet -- vertebrates	-0.036	-0.126	0.057	0.470
Migration	0.084	0.044	0.124	<0.001
Habitat -- open	0.111	0.059	0.168	<0.001
**Random effects**				
Phylogeny	0.632	0.510	0.786	
**Residuals**				
Residual	0.060	0.046	0.073	
**Summary statistics**				
DIC	1602.4			
R^2 estimate	0.963			

Supplementary Table 4. Predictors of range size for all birds calculated with a Bayesian phylogenetic mixed model (MCMCglmm). Variables with *p*-value estimates less than 0.05 are highlighted in grey. pMCMC represents double the probability that the coefficient estimate is above or below zero (whichever is smaller), based on the posterior distribution; CI = credible interval. Temperature = annual mean temperature. Temp. Var. = variation in monthly temperature values over a year (standard deviation). Precipitation = annual precipitation. Precip. Var. = variation in monthly precipitation values over a year (coefficient of variance). Lat. = median breeding range latitude (absolute value). Hemisphere – S. = whether the median breeding range latitude is found in the southern (1) or northern (0) hemisphere. Model includes migration data from BirdLife International; kiwis (order Apterygiformes) have been omitted.

**Fixed effects**	*z*-score	lower 95% CI	upper 95% CI	pMCMC
HWI	0.126	0.092	0.162	<0.001
Migration	0.474	0.416	0.524	<0.001
Mass	-0.012	-0.060	0.029	0.596
Island	-0.206	-0.226	-0.184	<0.001
Latitude	-0.269	-0.318	-0.220	<0.001
Hemisphere – S.	0.183	0.147	0.223	<0.001
Temperature	0.282	0.247	0.320	<0.001
Temp. Var.	0.861	0.817	0.908	<0.001
Precipitation	0.162	0.136	0.191	<0.001
Precip. Var.	-0.023	-0.048	0.000	0.050
Diet -- invertebrates	0.041	-0.033	0.117	0.274
Diet -- nectar	-0.033	-0.185	0.135	0.670
Diet -- omnivores	0.074	0.004	0.150	0.056
Diet -- plants	0.015	-0.120	0.160	0.818
Diet -- scavenger	0.391	0.075	0.716	0.016
Diet -- seeds	0.051	-0.052	0.137	0.280
Diet -- vertebrates	0.237	0.111	0.364	0.002
Habitat -- open	0.104	0.048	0.154	<0.001
Lat.*Hemisphere	-0.083	-0.126	-0.034	<0.001
**Random effects**				
Phylogeny	0.359	0.268	0.435	
**Residuals**				
Residual	0.464	0.447	0.479	
**Summary statistics**				
DIC	20110.8			
R^2 estimate	0.896			

Supplementary Table 5. Predictors of range size for passerines calculated with a Bayesian phylogenetic mixed model (MCMCglmm). Variables with *p*-value estimates less than 0.05 are highlighted in grey. pMCMC represents double the probability that the coefficient estimate is above or below zero (whichever is smaller), based on the posterior distribution; CI = credible interval. Temperature = annual mean temperature. Temp. Var. = variation in monthly temperature values over a year (standard deviation). Precipitation = annual precipitation. Precip. Var. = variation in monthly precipitation values over a year (coefficient of variance). Lat. = median breeding range latitude (absolute value). Hemisphere – S. = whether the median breeding range latitude is found in the southern (1) or northern (0) hemisphere. Model includes migration data from BirdLife International; kiwis (order Apterygiformes) have been omitted.

**Fixed effects**	*z*-score	lower 95% CI	upper 95% CI	pMCMC
HWI	0.150	0.118	0.184	<0.001
Migration	-0.010	-0.106	0.094	0.860
Mass	-0.032	-0.068	0.000	0.060
Island	-0.196	-0.223	-0.171	<0.001
Latitude	-0.262	-0.327	-0.197	<0.001
Hemisphere – S.	0.141	0.086	0.196	<0.001
Temperature	0.335	0.290	0.377	<0.001
Temp. Var.	1.070	1.000	1.130	<0.001
Precipitation	0.215	0.178	0.255	<0.001
Precip. Var.	-0.050	-0.082	-0.019	0.002
Diet -- invertebrates	0.061	-0.043	0.150	0.238
Diet -- nectar	-0.083	-0.294	0.122	0.418
Diet -- omnivores	0.112	0.022	0.212	0.022
Diet -- plants	0.131	-0.280	0.463	0.502
Diet -- scavenger	-0.461	-1.445	0.711	0.390
Diet -- seeds	0.098	-0.036	0.237	0.172
Diet -- vertebrates	0.431	-0.069	0.959	0.094
Habitat -- open	0.052	-0.010	0.116	0.126
Lat.*Hemisphere	-0.072	-0.127	-0.012	0.018
**Random effects**				
Phylogeny	0.275	0.195	0.348	
**Residuals**				
Residual	0.494	0.469	0.516	
**Summary statistics**				
DIC	12324.9			
R^2 estimate	0.896			

Supplementary Table 6. Predictors of range size for non-passerines calculated with a Bayesian phylogenetic mixed model (MCMCglmm). Variables with *p*-value estimates less than 0.05 are highlighted in grey. pMCMC represents double the probability that the coefficient estimate is above or below zero (whichever is smaller), based on the posterior distribution; CI = credible interval. Temperature = annual mean temperature. Temp. Var. = variation in monthly temperature values over a year (standard deviation). Precipitation = annual precipitation. Precip. Var. = variation in monthly precipitation values over a year (coefficient of variance). Lat. = median breeding range latitude (absolute value). Hemisphere – S. = whether the median breeding range latitude is found in the southern (1) or northern (0) hemisphere. Model includes migration data from BirdLife International; kiwis (order Apterygiformes) have been omitted.

**Fixed effects**	*z*-score	lower 95% CI	upper 95% CI	pMCMC
HWI	0.068	0.015	0.117	0.006
Migration	0.471	0.391	0.553	<0.001
Mass	-0.002	-0.058	0.056	0.980
Island	-0.208	-0.241	-0.175	<0.001
Latitude	-0.259	-0.337	-0.179	<0.001
Hemisphere – S.	0.209	0.150	0.266	<0.001
Temperature	0.208	0.149	0.273	<0.001
Temp. Var.	0.728	0.655	0.798	<0.001
Precipitation	0.118	0.074	0.163	<0.001
Precip. Var.	-0.013	-0.052	0.023	0.490
Diet -- invertebrates	0.031	-0.100	0.154	0.624
Diet -- nectar	-0.061	-0.330	0.208	0.626
Diet -- omnivores	0.035	-0.084	0.140	0.550
Diet -- plants	-0.033	-0.207	0.122	0.706
Diet -- scavenger	0.423	0.068	0.738	0.010
Diet -- seeds	0.015	-0.131	0.165	0.832
Diet -- vertebrates	0.224	0.073	0.378	0.008
Habitat -- open	0.202	0.105	0.287	<0.001
Lat.*Hemisphere	-0.138	-0.207	-0.068	<0.001
**Random effects**				
Phylogeny	0.246	0.172	0.334	
**Residuals**				
Residual	0.448	0.423	0.474	
**Summary statistics**				
DIC	8012.0			
R^2 estimate	0.892			

Supplementary Table 7. Predictors of migration for all birds calculated with a Bayesian phylogenetic mixed model (MCMCglmm), with migration as a binary response (logistic regression). Variables with *p*-value estimates less than 0.05 are highlighted in grey. pMCMC represents double the probability that the coefficient estimate is above or below zero (whichever is smaller), based on the posterior distribution; CI = credible interval. Temperature = annual mean temperature. Temp. Var. = variation in monthly temperature values over a year (standard deviation). Precipitation = annual precipitation. Precip. Var. = variation in monthly precipitation values over a year (coefficient of variance). Lat. = median breeding range latitude (absolute value). Hemisphere – S. = whether the median breeding range latitude is found in the southern (1) or northern (0) hemisphere. Model includes migration data from BirdLife International; kiwis (order Apterygiformes) have been omitted.

**Fixed effects**	*z*-score	lower 95% CI	upper 95% CI	pMCMC
HWI	2.279	1.887	2.613	<0.001
Mass	-0.843	-1.275	-0.511	<0.001
Island	-0.175	-0.387	0.031	0.094
Latitude	1.092	0.722	1.498	<0.001
Hemisphere – S.	0.035	-0.301	0.385	0.844
Temperature	0.097	-0.188	0.363	0.520
Temp. Var.	2.064	1.698	2.467	<0.001
Precipitation	0.369	0.079	0.636	0.014
Precip. Var.	-0.412	-0.638	-0.163	<0.001
Diet -- invertebrates	-0.048	-0.782	0.631	0.902
Diet -- nectar	-0.222	-1.799	1.509	0.786
Diet -- omnivores	-0.106	-0.831	0.520	0.748
Diet -- plants	1.069	-0.064	2.144	0.068
Diet -- scavenger	0.631	-1.481	2.585	0.548
Diet -- seeds	0.506	-0.322	1.333	0.214
Diet -- vertebrates	-0.369	-1.261	0.660	0.480
Habitat -- open	-0.044	-0.441	0.338	0.816
Lat.*Hemisphere	-0.170	-0.575	0.159	0.362
**Random effects**				
Phylogeny	24.1	15.6	33.4	
**Residuals**				
Residual	1	1	1	
**Summary statistics**				
DIC	3578.5			
R^2 estimate	0.890			

Supplementary Table 8. Predictors of migration for passerines calculated with a Bayesian phylogenetic mixed model (MCMCglmm), with migration as a binary response (logistic regression). Variables with *p*-value estimates less than 0.05 are highlighted in grey. pMCMC represents double the probability that the coefficient estimate is above or below zero (whichever is smaller), based on the posterior distribution; CI = credible interval. Temperature = annual mean temperature. Temp. Var. = variation in monthly temperature values over a year (standard deviation). Precipitation = annual precipitation. Precip. Var. = variation in monthly precipitation values over a year (coefficient of variance). Lat. = median breeding range latitude (absolute value). Hemisphere – S. = whether the median breeding range latitude is found in the southern (1) or northern (0) hemisphere. Model includes migration data from BirdLife International; kiwis (order Apterygiformes) have been omitted.

**Fixed effects**	*z*-score	lower 95% CI	upper 95% CI	pMCMC
HWI	2.167	1.712	2.633	<0.001
Mass	-0.974	-1.352	-0.578	<0.001
Island	-0.284	-0.655	0.056	0.114
Latitude	1.795	1.090	2.512	<0.001
Hemisphere – S.	0.927	0.300	1.528	0.002
Temperature	0.608	0.210	1.027	0.004
Temp. Var.	2.787	2.069	3.556	<0.001
Precipitation	0.624	0.153	1.151	0.018
Precip. Var.	-0.438	-0.809	-0.043	0.020
Diet -- invertebrates	-0.371	-1.473	0.579	0.524
Diet -- nectar	-1.470	-4.141	1.104	0.250
Diet -- omnivores	-0.478	-1.601	0.544	0.386
Diet -- plants	1.358	-1.162	4.049	0.306
Diet -- scavenger	-1.854	-8.687	4.093	0.620
Diet -- seeds	-0.104	-1.522	1.131	0.888
Diet -- vertebrates	1.141	-2.258	5.213	0.542
Habitat -- open	-0.465	-1.065	0.092	0.120
Lat.*Hemisphere	-0.007	-0.601	0.585	0.978
**Random effects**				
Phylogeny	27.1	14.8	41.7	
**Residuals**				
Residual	1	1	1	
**Summary statistics**				
DIC	1631.8			
R^2 estimate	0.913			

Supplementary Table 9. Predictors of migration for non-passerines calculated with a Bayesian phylogenetic mixed model (MCMCglmm), with migration as a binary response (logistic regression). Variables with *p*-value estimates less than 0.05 are highlighted in grey. pMCMC represents double the probability that the coefficient estimate is above or below zero (whichever is smaller), based on the posterior distribution; CI = credible interval. Temperature = annual mean temperature. Temp. Var. = variation in monthly temperature values over a year (standard deviation). Precipitation = annual precipitation. Precip. Var. = variation in monthly precipitation values over a year (coefficient of variance). Lat. = median breeding range latitude (absolute value). Hemisphere – S. = whether the median breeding range latitude is found in the southern (1) or northern (0) hemisphere. Model includes migration data from BirdLife International; kiwis (order Apterygiformes) have been omitted.

**Fixed effects**	*z*-score	lower 95% CI	upper 95% CI	pMCMC
HWI	1.682	1.160	2.209	<0.001
Mass	-0.404	-0.905	0.014	0.072
Island	-0.153	-0.400	0.102	0.242
Latitude	0.586	0.049	1.098	0.028
Hemisphere – S.	-0.593	-1.015	-0.135	0.006
Temperature	-0.587	-1.043	-0.125	0.012
Temp. Var.	1.359	0.883	1.898	<0.001
Precipitation	0.132	-0.221	0.510	0.496
Precip. Var.	-0.260	-0.596	0.056	0.128
Diet -- invertebrates	0.163	-1.107	1.249	0.764
Diet -- nectar	1.156	-1.327	3.664	0.346
Diet -- omnivores	0.159	-0.806	1.170	0.752
Diet -- plants	0.924	-0.437	2.325	0.196
Diet -- scavenger	0.653	-1.537	2.708	0.534
Diet -- seeds	1.098	-0.042	2.306	0.052
Diet -- vertebrates	-0.285	-1.510	0.873	0.614
Habitat -- open	0.500	-0.129	1.118	0.098
Lat.*Hemisphere	-0.420	-0.982	0.020	0.088
**Random effects**				
Phylogeny	16.0	8.3	24.7	
**Residuals**				
Residual	1	1	1	
**Summary statistics**				
DIC	1858.9			
R^2 estimate	0.864			

Supplementary Table 10. Predictors of HWI for all birds calculated using alternate values of migration in a Bayesian phylogenetic mixed model (MCMCglmm). Variables with *p*-value estimates less than 0.05 are highlighted in grey. pMCMC represents double the probability that the coefficient estimate is above or below zero (whichever is smaller), based on the posterior distribution; CI = credible interval. Temperature = annual mean temperature. Temp. Var. = variation in monthly temperature values over a year (standard deviation). Precipitation = annual precipitation. Precip. Var. = variation in monthly precipitation values over a year (coefficient of variance). Lat. = median breeding range latitude (absolute value). These models use migration data from Tobias *et al*. 2016 (A) and Eyres *et al*. 2017 (B). Kiwis (order Apterygiformes) have been omitted. C.f. Supplementary Table 1.

	**A**				**B**			
**Fixed effects**	*z*-score	lower 95% CI	upper 95% CI	pMCMC	*z*-score	lower 95% CI	upper 95% CI	pMCMC
Mass	-0.035	-0.068	-0.005	0.022	-0.032	-0.061	0.003	0.054
Island	0.014	0.003	0.024	0.012	0.013	0.002	0.023	0.008
Latitude	0.034	0.014	0.053	<0.001	0.029	0.011	0.047	<0.001
Temperature	0.029	0.009	0.048	0.008	0.023	0.003	0.040	0.022
Temp. Var.	0.117	0.095	0.137	<0.001	0.112	0.089	0.134	<0.001
Precipitation	0.022	0.009	0.037	<0.001	0.022	0.009	0.036	0.004
Precip. Var.	-0.022	-0.033	-0.011	<0.001	-0.021	-0.033	-0.010	0.002
Territoriality	-0.122	-0.155	-0.096	<0.001	-0.123	-0.150	-0.095	<0.001
Diet -- invertebrates	-0.053	-0.095	-0.010	0.012	-0.058	-0.099	-0.016	0.006
Diet -- nectar	0.092	0.012	0.186	0.040	0.088	-0.005	0.178	0.050
Diet -- omnivores	-0.035	-0.074	0.005	0.092	-0.041	-0.080	-0.002	0.048
Diet -- plants	-0.007	-0.084	0.078	0.858	-0.017	-0.109	0.059	0.664
Diet -- scavenger	0.068	-0.102	0.282	0.478	0.047	-0.132	0.229	0.634
Diet -- seeds	-0.017	-0.070	0.040	0.540	-0.025	-0.077	0.031	0.368
Diet -- vertebrates	-0.067	-0.140	0.005	0.080	-0.070	-0.142	0.003	0.048
Migration	0.158	0.122	0.196	<0.001	0.140	0.111	0.167	<0.001
Habitat -- open	0.061	0.031	0.090	<0.001	0.056	0.032	0.087	<0.001
**Random effects**								
Phylogeny	0.705	0.588	0.846		0.706	0.577	0.835	
**Residuals**								
Residual	0.061	0.055	0.068		0.061	0.055	0.067	
**Summary statistics**								
DIC	4412.4				4385.9			
R^2 estimate	0.964				0.964			

Supplementary Table 11. Predictors of HWI for passerines calculated using alternate values of migration in a Bayesian phylogenetic mixed model (MCMCglmm). Variables with *p*-value estimates less than 0.05 are highlighted in grey. pMCMC represents double the probability that the coefficient estimate is above or below zero (whichever is smaller), based on the posterior distribution; CI = credible interval. Temperature = annual mean temperature. Temp. Var. = variation in monthly temperature values over a year (standard deviation). Precipitation = annual precipitation. Precip. Var. = variation in monthly precipitation values over a year (coefficient of variance). Lat. = median breeding range latitude (absolute value). These models use migration data from Tobias *et al*. 2016 (A) and Eyres *et al*. 2017 (B). Kiwis (order Apterygiformes) have been omitted. C.f. Supplementary Table 2.

	A				B			
**Fixed effects**	*z*-score	lower 95% CI	upper 95% CI	pMCMC	*z*-score	lower 95% CI	upper 95% CI	pMCMC
Mass	0.001	-0.035	0.033	0.950	0.005	-0.028	0.039	0.788
Island	0.022	0.001	0.042	0.028	0.021	0.002	0.041	0.046
Latitude	0.053	0.021	0.083	0.004	0.044	0.011	0.077	0.012
Temperature	0.011	-0.022	0.042	0.548	0.002	-0.032	0.036	0.940
Temp. Var.	0.168	0.129	0.211	<0.001	0.157	0.113	0.199	<0.001
Precipitation	0.077	0.054	0.103	<0.001	0.077	0.053	0.105	<0.001
Precip. Var.	-0.041	-0.063	-0.021	<0.001	-0.039	-0.059	-0.017	<0.001
Territoriality	-0.175	-0.217	-0.130	<0.001	-0.170	-0.214	-0.121	<0.001
Diet -- invertebrates	-0.088	-0.160	0.003	0.040	-0.099	-0.185	-0.028	0.010
Diet -- nectar	0.155	0.001	0.319	0.044	0.148	-0.001	0.301	0.072
Diet -- omnivores	-0.103	-0.178	-0.028	0.010	-0.116	-0.190	-0.047	<0.001
Diet -- plants	-0.257	-0.488	0.016	0.060	-0.326	-0.575	-0.059	0.010
Diet -- scavenger	0.065	-0.593	0.782	0.904	0.144	-0.562	0.782	0.710
Diet -- seeds	-0.102	-0.186	0.011	0.052	-0.110	-0.202	-0.003	0.030
Diet -- vertebrates	-0.298	-0.638	0.068	0.100	-0.285	-0.670	0.061	0.118
Migration	0.268	0.201	0.337	<0.001	0.267	0.206	0.321	<0.001
Habitat -- open	0.062	0.014	0.109	0.016	0.049	0.005	0.102	0.052
**Random effects**								
Phylogeny	0.968	0.837	1.106		0.969	0.842	1.115	
**Residuals**								
Residual	0.120	0.110	0.133		0.118	0.107	0.131	
**Summary statistics**								
DIC	6423.0				6229.7			
R^2 estimate	0.960				0.963			

Supplementary Table 12. Predictors of HWI for non-passerines calculated using alternate values of migration in a Bayesian phylogenetic mixed model (MCMCglmm). Variables with *p*-value estimates less than 0.05 are highlighted in grey. pMCMC represents double the probability that the coefficient estimate is above or below zero (whichever is smaller), based on the posterior distribution; CI = credible interval. Temperature = annual mean temperature. Temp. Var. = variation in monthly temperature values over a year (standard deviation). Precipitation = annual precipitation. Precip. Var. = variation in monthly precipitation values over a year (coefficient of variance). Lat. = median breeding range latitude (absolute value). These models use migration data from Tobias *et al*. 2016 (A) and Eyres *et al*. 2017 (B). Kiwis (order Apterygiformes) have been omitted. C.f. Supplementary Table 3.

	**A**				**B**			
**Fixed effects**	*z*-score	lower 95% CI	upper 95% CI	pMCMC	*z*-score	lower 95% CI	upper 95% CI	pMCMC
Mass	-0.077	-0.126	-0.033	0.002	-0.075	-0.119	-0.026	0.002
Island	0.012	-0.003	0.029	0.154	0.012	-0.005	0.029	0.168
Latitude	0.037	0.008	0.069	0.026	0.032	0.004	0.062	0.034
Temperature	0.047	0.016	0.080	0.006	0.041	0.012	0.077	0.014
Temp. Var.	0.099	0.066	0.130	<0.001	0.098	0.066	0.128	<0.001
Precipitation	-0.035	-0.058	-0.011	0.006	-0.034	-0.057	-0.012	0.006
Precip. Var.	-0.003	-0.020	0.018	0.808	-0.003	-0.022	0.017	0.780
Territoriality	-0.117	-0.165	-0.060	<0.001	-0.116	-0.178	-0.067	<0.001
Diet -- invertebrates	-0.058	-0.140	0.027	0.186	-0.055	-0.124	0.034	0.198
Diet -- nectar	0.035	-0.133	0.176	0.622	0.042	-0.112	0.186	0.578
Diet -- omnivores	0.018	-0.049	0.084	0.620	0.019	-0.046	0.077	0.578
Diet -- plants	0.062	-0.039	0.160	0.250	0.060	-0.033	0.158	0.236
Diet -- scavenger	0.062	-0.151	0.283	0.602	0.052	-0.152	0.267	0.628
Diet -- seeds	0.064	-0.027	0.140	0.162	0.058	-0.031	0.147	0.212
Diet -- vertebrates	-0.043	-0.131	0.061	0.392	-0.042	-0.130	0.055	0.372
Migration	0.092	0.039	0.148	0.002	0.073	0.035	0.114	<0.001
Habitat -- open	0.114	0.061	0.170	<0.001	0.115	0.056	0.165	<0.001
**Random effects**								
Phylogeny	0.623	0.501	0.765		0.627	0.504	0.776	
**Residuals**								
Residual	0.061	0.048	0.074		0.062	0.050	0.076	
**Summary statistics**								
DIC	1785.9				1873.3			
R^2 estimate	0.963				0.962			

Supplementary Table 13. Predictors of range size for all birds calculated using alternate values of migration in a Bayesian phylogenetic mixed model (MCMCglmm). Variables with *p*-value estimates less than 0.05 are highlighted in grey. pMCMC represents double the probability that the coefficient estimate is above or below zero (whichever is smaller), based on the posterior distribution; CI = credible interval. Temperature = annual mean temperature. Temp. Var. = variation in monthly temperature values over a year (standard deviation). Precipitation = annual precipitation. Precip. Var. = variation in monthly precipitation values over a year (coefficient of variance). Lat. = median breeding range latitude (absolute value). Hemisphere – S. = whether the median breeding range latitude is found in the southern (1) or northern (0) hemisphere. Models use migration data from Tobias *et al*. 2016 (A) and Eyres *et al*. 2017 (B). Kiwis (order Apterygiformes) have been omitted. C.f. Supplementary Table 4.

	A				B			
**Fixed effects**	*z*-score	lower 95% CI	upper 95% CI	pMCMC	*z*-score	lower 95% CI	upper 95% CI	pMCMC
HWI	0.166	0.131	0.199	<0.001	0.135	0.100	0.165	<0.001
Migration	0.047	-0.024	0.122	0.214	0.437	0.381	0.500	<0.001
Mass	-0.023	-0.061	0.021	0.270	-0.013	-0.054	0.028	0.566
Island	-0.207	-0.230	-0.185	<0.001	-0.207	-0.231	-0.188	<0.001
Latitude	-0.230	-0.278	-0.176	<0.001	-0.269	-0.317	-0.221	<0.001
Hemisphere – S.	0.162	0.128	0.209	<0.001	0.199	0.162	0.237	<0.001
Temperature	0.288	0.250	0.326	<0.001	0.287	0.251	0.327	<0.001
Temp. Var.	0.922	0.875	0.973	<0.001	0.868	0.823	0.920	<0.001
Precipitation	0.184	0.154	0.213	<0.001	0.166	0.138	0.196	<0.001
Precip. Var.	-0.046	-0.072	-0.024	<0.001	-0.027	-0.050	0.002	0.044
Diet -- invertebrates	0.049	-0.033	0.121	0.228	0.043	-0.037	0.118	0.280
Diet -- nectar	-0.058	-0.212	0.128	0.470	-0.048	-0.201	0.104	0.558
Diet -- omnivores	0.078	0.005	0.155	0.044	0.077	0.008	0.152	0.032
Diet -- plants	0.027	-0.115	0.183	0.756	0.042	-0.102	0.178	0.524
Diet -- scavenger	0.427	0.108	0.748	0.012	0.412	0.115	0.764	0.016
Diet -- seeds	0.072	-0.024	0.170	0.162	0.070	-0.026	0.162	0.162
Diet -- vertebrates	0.225	0.111	0.353	0.004	0.225	0.102	0.348	<0.001
Habitat -- open	0.106	0.056	0.155	<0.001	0.110	0.056	0.162	<0.001
Lat.*Hemisphere	-0.102	-0.147	-0.057	<0.001	-0.068	-0.111	-0.026	0.004
**Random effects**								
Phylogeny	0.352	0.270	0.447		0.342	0.255	0.429	
**Residuals**								
Residual	0.476	0.460	0.493		0.468	0.453	0.488	
**Summary statistics**								
DIC	20344.4				20064.6			
R^2 estimate	0.888				0.896			

Supplementary Table 14. Predictors of range size for passerines calculated using alternate values of migration in a Bayesian phylogenetic mixed model (MCMCglmm). Variables with *p*-value estimates less than 0.05 are highlighted in grey. pMCMC represents double the probability that the coefficient estimate is above or below zero (whichever is smaller), based on the posterior distribution; CI = credible interval. Variables with *p*-values less than 0.05 are highlighted in grey. Temperature = annual mean temperature. Temp. Var. = variation in monthly temperature values over a year (standard deviation). Precipitation = annual precipitation. Precip. Var. = variation in monthly precipitation values over a year (coefficient of variance). Lat. = median breeding range latitude (absolute value). Hemisphere – S. = whether the median breeding range latitude is found in the southern (1) or northern (0) hemisphere. These models use migration data from Tobias *et al*. 2016 (A) and Eyres *et al*. 2017 (B). Kiwis (order Apterygiformes) have been omitted. C.f. Supplementary Table 5.

	**A**				**B**			
**Fixed effects**	*z*-score	lower 95% CI	upper 95% CI	pMCMC	*z*-score	lower 95% CI	upper 95% CI	pMCMC
HWI	0.150	0.118	0.184	<0.001	0.125	0.088	0.156	<0.001
Migration	-0.010	-0.106	0.094	0.860	0.419	0.329	0.497	<0.001
Mass	-0.032	-0.068	0.000	0.060	-0.024	-0.056	0.016	0.194
Island	-0.196	-0.223	-0.171	<0.001	-0.195	-0.226	-0.168	<0.001
Latitude	-0.262	-0.327	-0.197	<0.001	-0.314	-0.381	-0.251	<0.001
Hemisphere – S.	0.141	0.086	0.196	<0.001	0.175	0.120	0.229	<0.001
Temperature	0.335	0.290	0.377	<0.001	0.337	0.294	0.382	<0.001
Temp. Var.	1.070	1.000	1.130	<0.001	1.019	0.951	1.089	<0.001
Precipitation	0.215	0.178	0.255	<0.001	0.194	0.158	0.233	<0.001
Precip. Var.	-0.050	-0.082	-0.019	0.002	-0.031	-0.065	-0.004	0.036
Diet -- invertebrates	0.061	-0.043	0.150	0.238	0.053	-0.037	0.155	0.292
Diet -- nectar	-0.083	-0.294	0.122	0.418	-0.060	-0.278	0.145	0.604
Diet -- omnivores	0.112	0.022	0.212	0.022	0.109	0.005	0.210	0.046
Diet -- plants	0.131	-0.280	0.463	0.502	0.117	-0.271	0.467	0.546
Diet -- scavenger	-0.461	-1.445	0.711	0.390	-0.384	-1.400	0.660	0.454
Diet -- seeds	0.098	-0.036	0.237	0.172	0.098	-0.024	0.231	0.130
Diet -- vertebrates	0.431	-0.069	0.959	0.094	0.352	-0.191	0.782	0.172
Habitat -- open	0.052	-0.010	0.116	0.126	0.059	-0.014	0.122	0.100
Lat.*Hemisphere	-0.072	-0.127	-0.012	0.018	-0.031	-0.094	0.022	0.288
**Random effects**								
Phylogeny	0.275	0.195	0.348		0.266	0.189	0.349	
**Residuals**								
Residual	0.494	0.469	0.516		0.488	0.462	0.509	
**Summary statistics**								
DIC	12324.9				12067.8			
R^2 estimate	0.896				0.899			

Supplementary Table 15. Predictors of range size for non-passerines calculated using alternate values of migration in a Bayesian phylogenetic mixed model (MCMCglmm). Variables with *p*-value estimates less than 0.05 are highlighted in grey. pMCMC represents double the probability that the coefficient estimate is above or below zero (whichever is smaller), based on the posterior distribution; CI = credible interval. Temperature = annual mean temperature. Temp. Var. = variation in monthly temperature values over a year (standard deviation). Precipitation = annual precipitation. Precip. Var. = variation in monthly precipitation values over a year (coefficient of variance). Lat. = median breeding range latitude (absolute value). Hemisphere – S. = whether the median breeding range latitude is found in the southern (1) or northern (0) hemisphere. These models use migration data from Tobias *et al*. 2016 (A) and Eyres *et al*. 2017 (B). Kiwis (order Apterygiformes) have been omitted. C.f. Supplementary Table 6.

	A				B			
**Fixed effects**	*z*-score	lower 95% CI	upper 95% CI	pMCMC	*z*-score	lower 95% CI	upper 95% CI	pMCMC
HWI	0.100	0.046	0.146	<0.001	0.077	0.031	0.131	0.002
Migration	0.084	-0.015	0.205	0.110	0.451	0.367	0.534	<0.001
Mass	-0.007	-0.060	0.057	0.834	0.002	-0.051	0.059	0.986
Island	-0.209	-0.240	-0.177	<0.001	-0.211	-0.244	-0.178	<0.001
Latitude	-0.237	-0.310	-0.147	<0.001	-0.263	-0.344	-0.190	<0.001
Hemisphere – S.	0.179	0.118	0.242	<0.001	0.220	0.162	0.279	<0.001
Temperature	0.203	0.135	0.269	<0.001	0.203	0.137	0.264	<0.001
Temp. Var.	0.779	0.710	0.843	<0.001	0.723	0.655	0.795	<0.001
Precipitation	0.137	0.092	0.185	<0.001	0.120	0.074	0.159	<0.001
Precip. Var.	-0.031	-0.073	0.005	0.108	-0.011	-0.047	0.028	0.552
Diet -- invertebrates	0.035	-0.101	0.161	0.600	0.031	-0.085	0.168	0.634
Diet -- nectar	-0.031	-0.321	0.219	0.818	-0.025	-0.283	0.236	0.864
Diet -- omnivores	0.044	-0.067	0.155	0.432	0.048	-0.062	0.156	0.414
Diet -- plants	-0.025	-0.198	0.147	0.784	-0.009	-0.184	0.144	0.896
Diet -- scavenger	0.458	0.103	0.782	0.010	0.438	0.108	0.758	0.008
Diet -- seeds	0.064	-0.092	0.213	0.414	0.062	-0.075	0.215	0.412
Diet -- vertebrates	0.206	0.045	0.359	0.008	0.214	0.066	0.360	0.010
Habitat -- open	0.215	0.125	0.312	<0.001	0.219	0.124	0.310	<0.001
Lat.*Hemisphere	-0.155	-0.225	-0.082	<0.001	-0.126	-0.202	-0.063	<0.001
**Random effects**								
Phylogeny	0.262	0.179	0.356		0.252	0.166	0.349	
**Residuals**								
Residual	0.460	0.437	0.490		0.450	0.426	0.475	
**Summary statistics**								
DIC	8104.2				8075.4			
R^2 estimate	0.883				0.892			

Supplementary Table 16. Predictors of migration for all birds calculated using alternate values of migration in a Bayesian phylogenetic mixed model (MCMCglmm) with migration as a binary response (logistic regression). Variables with *p*-value estimates less than 0.05 are highlighted in grey. pMCMC represents double the probability that the coefficient estimate is above or below zero (whichever is smaller), based on the posterior distribution; CI = credible interval. Temperature = annual mean temperature. Temp. Var. = variation in monthly temperature values over a year (standard deviation). Precipitation = annual precipitation. Precip. Var. = variation in monthly precipitation values over a year (coefficient of variance). Lat. = median breeding range latitude (absolute value). Hemisphere – S. = whether the median breeding range latitude is found in the southern (1) or northern (0) hemisphere. These models use migration data from Tobias *et al*. 2016 (A) and Eyres *et al*. 2017 (B). Kiwis (order Apterygiformes) have been omitted. C.f. Supplementary Table 7.

	**A**				**B**			
**Fixed effects**	*z*-score	lower 95% CI	upper 95% CI	pMCMC	*z*-score	lower 95% CI	upper 95% CI	pMCMC
HWI	2.981	2.449	3.572	<0.001	2.484	2.049	2.869	<0.001
Mass	-1.202	-1.735	-0.746	<0.001	-1.113	-1.464	-0.736	<0.001
Island	-0.172	-0.488	0.142	0.292	-0.037	-0.244	0.178	0.744
Latitude	1.233	0.751	1.760	<0.001	0.918	0.548	1.300	<0.001
Hemisphere – S.	-0.644	-1.164	-0.038	0.034	-0.598	-0.941	-0.247	<0.001
Temperature	-0.118	-0.512	0.311	0.532	0.026	-0.276	0.295	0.838
Temp. Var.	1.529	1.073	1.998	<0.001	2.091	1.701	2.480	<0.001
Precipitation	0.500	0.087	0.931	0.018	0.261	-0.033	0.552	0.086
Precip. Var.	-0.405	-0.739	-0.050	0.012	-0.262	-0.499	-0.021	0.024
Diet -- invertebrates	1.240	-0.007	2.552	0.048	0.354	-0.363	1.193	0.354
Diet -- nectar	0.773	-1.762	3.534	0.538	-0.369	-2.239	1.392	0.692
Diet -- omnivores	0.701	-0.539	2.066	0.296	-0.060	-0.729	0.783	0.876
Diet -- plants	0.529	-1.090	2.516	0.590	0.651	-0.551	1.829	0.308
Diet -- scavenger	0.026	-3.360	3.542	0.978	1.091	-0.775	3.398	0.314
Diet -- seeds	0.957	-0.515	2.361	0.232	-0.156	-0.978	0.833	0.716
Diet -- vertebrates	1.884	0.397	3.351	0.016	0.175	-0.824	1.111	0.740
Habitat -- open	-0.676	-1.163	-0.124	0.008	0.026	-0.408	0.436	0.912
Lat.*Hemisphere	-0.324	-0.844	0.145	0.182	-0.064	-0.432	0.258	0.710
**Random effects**								
Phylogeny	27.6	16.9	39.3		18.8	11.9	25.9	
**Residuals**								
Residual	1	1	1		1	1	1	
**Summary statistics**								
DIC	1972.1				3163.8			
R^2 estimate	0.910				0.873			

Supplementary Table 17. Predictors of migration for passerines calculated using alternate values of migration in a Bayesian phylogenetic mixed model (MCMCglmm) with migration as a binary response (logistic regression). Variables with *p*-value estimates less than 0.05 are highlighted in grey. pMCMC represents double the probability that the coefficient estimate is above or below zero (whichever is smaller), based on the posterior distribution; CI = credible interval. Temperature = annual mean temperature. Temp. Var. = variation in monthly temperature values over a year (standard deviation). Precipitation = annual precipitation. Precip. Var. = variation in monthly precipitation values over a year (coefficient of variance). Lat. = median breeding range latitude (absolute value). Hemisphere – S. = whether the median breeding range latitude is found in the southern (1) or northern (0) hemisphere. Models use migration data from Tobias *et al*. 2016 (A) and Eyres *et al*. 2017 (B). Kiwis (order Apterygiformes) have been omitted. C.f. Supplementary Table 8.

	**A**				**B**			
**Fixed effects**	*z*-score	lower 95% CI	upper 95% CI	pMCMC	*z*-score	lower 95% CI	upper 95% CI	pMCMC
HWI	2.211	1.672	2.787	<0.001	2.507	1.946	3.067	<0.001
Mass	-1.181	-1.671	-0.708	<0.001	-1.049	-1.530	-0.625	<0.001
Island	-0.445	-0.906	-0.008	0.046	-0.126	-0.486	0.231	0.492
Latitude	1.854	1.067	2.607	<0.001	1.841	1.076	2.482	<0.001
Hemisphere – S.	-0.031	-0.814	0.786	0.954	-0.223	-0.822	0.413	0.490
Temperature	0.216	-0.277	0.704	0.388	0.383	-0.031	0.814	0.080
Temp. Var.	1.516	0.895	2.147	<0.001	2.500	1.803	3.250	<0.001
Precipitation	0.576	-0.051	1.259	0.086	0.495	-0.102	0.977	0.074
Precip. Var.	-0.395	-0.838	0.110	0.106	-0.354	-0.763	0.043	0.082
Diet -- invertebrates	1.067	-0.349	2.625	0.160	0.168	-0.992	1.258	0.748
Diet -- nectar	-0.502	-4.038	3.375	0.826	-1.315	-4.216	1.205	0.368
Diet -- omnivores	0.807	-0.588	2.417	0.322	-0.104	-1.239	1.058	0.814
Diet -- plants	-4.462	-9.866	0.591	0.064	0.199	-2.819	2.945	0.892
Diet -- scavenger	-0.579	-8.798	6.809	0.924	-1.880	-8.605	4.449	0.636
Diet -- seeds	0.714	-1.028	2.342	0.400	-0.832	-2.071	0.616	0.244
Diet -- vertebrates	3.011	-0.169	6.587	0.076	1.934	-1.998	5.492	0.340
Habitat -- open	-0.969	-1.584	-0.307	0.002	-0.181	-0.789	0.425	0.566
Lat.*Hemisphere	-0.439	-1.151	0.262	0.248	-0.371	-0.941	0.238	0.246
**Random effects**								
Phylogeny	18.3	8.8	28.3		28.4	14.7	46.4	
**Residuals**								
Residual	1	1	1		1	1	1	
**Summary statistics**								
DIC	1040.0				1432.6			
R^2 estimate	0.902				0.913			

Supplementary Table 18. Predictors of migration for non-passerines calculated using alternate values of migration in a Bayesian phylogenetic mixed model (MCMCglmm) with migration as a binary response (logistic regression). Variables with *p*-value estimates less than 0.05 are highlighted in grey. pMCMC represents double the probability that the coefficient estimate is above or below zero (whichever is smaller), based on the posterior distribution; CI = credible interval. Temperature = annual mean temperature. Temp. Var. = variation in monthly temperature values over a year (standard deviation). Precipitation = annual precipitation. Precip. Var. = variation in monthly precipitation values over a year (coefficient of variance). Lat. = median breeding range latitude (absolute value). Hemisphere – S. = whether the median breeding range latitude is found in the southern (1) or northern (0) hemisphere. These models use migration data from Tobias *et al*. 2016 (A) and Eyres *et al*. 2017 (B). Kiwis (order Apterygiformes) have been omitted. C.f. Supplementary Table 9.

	**A**				**B**			
**Fixed effects**	*z*-score	lower 95% CI	upper 95% CI	pMCMC	*z*-score	lower 95% CI	upper 95% CI	pMCMC
HWI	3.202	2.276	4.391	<0.001	1.636	1.127	2.125	<0.001
Mass	-0.606	-1.371	0.044	0.088	-0.779	-1.224	-0.392	<0.001
Island	0.050	-0.380	0.484	0.826	-0.026	-0.293	0.255	0.870
Latitude	0.599	-0.172	1.463	0.138	0.281	-0.193	0.804	0.246
Hemisphere – S.	-1.280	-2.001	-0.444	<0.001	-0.829	-1.239	-0.400	<0.001
Temperature	-0.666	-1.414	-0.031	0.052	-0.439	-0.856	-0.072	0.038
Temp. Var.	1.417	0.792	2.179	<0.001	1.743	1.194	2.185	<0.001
Precipitation	0.428	-0.208	1.129	0.228	0.022	-0.378	0.372	0.918
Precip. Var.	-0.282	-0.817	0.222	0.292	-0.097	-0.400	0.237	0.548
Diet -- invertebrates	1.250	-1.092	3.667	0.316	0.670	-0.600	2.009	0.302
Diet -- nectar	2.342	-2.211	6.847	0.290	0.515	-2.352	3.304	0.710
Diet -- omnivores	0.212	-2.263	2.642	0.880	0.029	-1.232	1.256	0.914
Diet -- plants	0.853	-1.866	3.579	0.550	0.642	-0.861	2.095	0.404
Diet -- scavenger	-0.863	-4.624	2.836	0.668	0.926	-1.118	3.095	0.396
Diet -- seeds	1.219	-1.360	4.175	0.382	0.539	-0.714	1.967	0.388
Diet -- vertebrates	1.384	-1.082	3.911	0.288	0.342	-1.113	1.643	0.608
Habitat -- open	-0.021	-1.134	1.122	0.954	0.378	-0.242	0.949	0.246
Lat.*Hemisphere	-0.287	-1.048	0.463	0.430	0.084	-0.379	0.531	0.712
**Random effects**								
Phylogeny	30.2	14.4	49.5		8.8	4.6	13.5	
**Residuals**								
Residual	1	1	1		1	1	1	
**Summary statistics**								
DIC	882.3				1655.2			
R^2 estimate	0.917				0.814			

Supplementary Table 19. Correlations between predictor variables across all birds included in the main model. For pairs of continuous variables, or a continuous and a categorical variable, the adjusted R^2 is shown; for pairs of a binary variable and a categorical variable, a pseudo-R^2 is calculated as 1 – (residual deviance)/(null deviance), based on a logistic regression. R^2/pseudo-R^2 values greater than 0.5 have been highlighted in grey; cells with information repeated elsewhere on the table are marked in black. Migratory data is from BirdLife International. Temp. = annual mean temperature. Temp. Var. = variation in monthly temperature values over a year (standard deviation). Precip. = annual precipitation. Precip. Var. = variation in monthly precipitation values over a year (coefficient of variance).

	Island	*Latitude*	*Temp.*	*Temp. Var.*	*Precip.*	*Precip. Var.*	*Territoriality*	*Diet*	*Migration*	*Habitat*
Mass	0.008	0.026	0.016	0.011	0.005	0.011	0.000	0.369	0.023	0.088
Island		0.001	0.026	0.096	0.082	0.002	0.007	0.012	0.001	0.002
Latitude			0.724	0.671	0.331	0.275	0.035	0.071	0.303	0.119
Temperature				0.653	0.287	0.334	0.031	0.063	0.276	0.112
Temp. Var.					0.515	0.259	0.031	0.086	0.289	0.120
Precipitation						0.394	0.017	0.093	0.134	0.152
Precip. Var.							0.019	0.050	0.149	0.124
Territoriality								0.089	0.074	0.054
Diet									0.050	0.125
Migration										0.105

Supplementary Table 20. Variance inflation factors (VIFs) for the main models (based on migratory data from BirdLife International). A VIF = 1 indicates no correlation between the variable and any other predictor variable in the model; a VIF < 10 indicates low correlation. Greyed out cells indicate variable absence from model. Temp. Var. = variation in monthly temperature values over a year (standard deviation). Precip. Var. = variation in monthly precipitation values over a year (coefficient of variance). Lat. = median breeding range latitude (absolute value). Hemi. – S. = whether the median breeding range latitude is found in the southern (1) or northern (0) hemisphere. Within the dietary categories, invert. = invertebrates, omni. = omnivore, scav. = scavenger, and vert. = vertebrates.

Model:	HWI			Range size			Migration		
	All birds	Pass.	Non-pass.	All birds	Pass.	Non-pass.	All birds	Pass.	Non-pass.
See Table:	S1	S2	S3	S4	S5	S6	S7	S8	S9
Mass	1.72	1.15	2.28	1.74	1.14	2.48	1.74	1.13	2.48
Island	1.41	1.40	1.50	1.56	1.58	1.65	1.56	1.58	1.65
Latitude	5.30	5.32	5.85	10.18	10.14	10.83	10.10	10.01	10.80
Temperature	5.00	4.21	6.69	5.33	4.49	7.07	5.33	4.47	7.05
Temp. Var.	5.94	7.62	5.30	8.60	10.36	8.07	8.48	10.23	8.01
Precipitation	3.01	2.93	3.26	3.15	3.13	3.37	3.14	3.07	3.37
Precip. Var.	2.12	1.95	2.48	2.38	2.21	2.75	2.36	2.17	2.74
Territoriality	1.29	1.29	1.30						
Diet – invert.	3.15	3.13	2.79	2.98	2.98	2.70	2.98	2.97	2.69
Diet – nectar	1.55	1.22	2.40	1.74	1.22	2.54	1.72	1.22	2.53
Diet – omni.	2.00	2.15	1.86	1.99	2.16	1.87	1.99	2.16	1.87
Diet – plants	1.29	1.04	1.49	1.28	1.04	1.50	1.28	1.04	1.49
Diet – scav.	1.07	1.01	1.12	1.07	1.01	1.12	1.07	1.01	1.12
Diet – seeds	1.73	2.01	1.41	1.73	2.03	1.42	1.73	2.01	1.42
Diet – vert.	1.68	1.03	2.08	1.67	1.03	2.09	1.67	1.03	2.09
Migration	1.65	1.68	1.66	1.70	1.70	1.66			
Habitat – open	1.47	1.23	1.87	1.49	1.26	1.92	1.47	1.25	1.86
HWI				1.58	1.30	1.66	1.48	1.26	1.61
Hemi. – S.				1.46	1.47	1.51	1.45	1.45	1.51
Lat.*Hemi.				2.45	2.40	2.59	2.45	2.36	2.59

Supplementary Notes

For access to specimens, we gratefully acknowledge decades – and in some cases centuries – of work of countless curators, collections managers, collectors, and assistants associated with the following museums: Auckland War Memorial Museum, American Museum of Natural History, Universidad de los Andes, Academy of Natural Sciences Philadelphia, Australian National Wildlife Collection, Academia Sinica Institute of Zoology, Beijing Museum of Natural History, Colección Boliviana de Fauna, Carnegie Museum, Canterbury Museum, Centro de Ornitología y Biodiversidad, University of Copenhagen, Cornell University Museum of Vertebrates, Delaware Museum of Natural History, Durban Natural Science Museum, Edwin and Yoshika Willis collection, Florida Museum of Natural History, Miguel Lillo Foundation, Field Museum of Natural History, Universidad Nacional de Colombia: Museo de Historia Natural, National Institute of Amazonian Research, Royal Belgian Institute of Natural Sciences, University of Kansas Natural History Museum, Natural History Museum of Los Angeles County, Liverpool Museum, Louisiana State University: Museum of Natural Science, Bernardino Rivadavia Natural Sciences Argentine Museum, Museu de Biologia Professor Mello Leitão, Museum of Comparative Zoology: Harvard University, Moore Lab of Zoology: Occidental College, Manchester Metropolitan University Museum, National Museum of Natural History, Museo Nacional de Historia Natural y Antropología Uruguay, National Museum of Brazil Museu Paraense Emilio Goeldi, Natural History Museum of Giacomo Doria, Museum of Natural History: Lima, Bogor Zoology Museum, Museum of Zoology of the University of São Paulo, Natural History Museum Tring, Natural History Museum of Denmark, National Museums of Kenya, Sri Lanka National Museum, Oxford University Museum, Philippine National Museum, Royal Belgian Institute of Natural Sciences, Royal Museum for Central Africa, Naturalis Biodiversity Center, Royal Ontario Museum, Shanghai Natural History Museum, State Natural History Museum: Braunschweig, Smithsonian Tropical Research Institute, Ditsong National Museum of Natural History, Université d'Antananarivo: Département de Biologie Animale, University of British Columbia: Beaty Biodiversity Museum, Federal University of Pernambuco, University of Michigan: Museum of Zoology, University of the Philippines Los Baños Museum of Natural History, National Museum of Natural History: Smithsonian Institute, Burke Museum of Natural History and Culture, Western Australia Museum, Yale Peabody Museum of Natural History, Alexander Koenig Research Museum, Natural History Museum, and Lee Kong Chian Natural History Museum. For help measuring specimens, we thank: Abigail E Rothrauff, Ada Grabowska-Zhang, Alex Pigot, Ben Daly, Ben G Freeman, Bianca Darski, Bicheng Li, Brian Weeks, Camilla G Meneses, Chris Trisos, Clem Fisher, Daniel Swindlehurst, Delaney B Morrow, Dominic Chesire, Eden Cottee-Jones, Edson Mlamba, Elizabeth Derryberry, Elliot T Miller, F. Garry Stiles, Faansie Peacock, Frank Rheindt, Gabriel Jamie, Guilherme Brito, He Peng, Henrike Schulte To Buhne, Henry Pollack, Hevana Lima, Isabel Gomez, James M Maley, Jarome Ali, Jay P. McEntee, Jeffrey Tyler Howard, Jérôme Fuchs, Jon Fjeldsa, Juan Carlos Gonzalez, Junfu Wang, K. Eldridge, Kaiya L Provost, Kristof Zyskowski, Lankani Somarathna, Lincoln D. C. Fishpool, Luis Fabio Silveiro, M Rayner, M. C. Stoddard, M.P. Camelo, Marcus Chua, Maria Livrand, Martin Paeckert, Matheus Bitencourt, Maura Jurado, Michael Harvey, Michaela Forthuber, Milly Hong, Mmatjie Mashao, Natalia Garcia, Natasha Turner, Nate Rice, Nicolas Soulages, Nigel Collar, Olivier Pauwels, Oscar Johnson, P R Sweet, Peter Hosner, Phil Birget, Philip Chapman, R Paul Schofield, Rauri Bowie, Rebekah Mayhew, René Marie Lafontaine, Robert Lauer, Robert People, Robert Ricklefs, Rolly C. Urriza, Ron Johnstone, Rosalbina Butron Loayza, Ross Crates, Sarah Rosenberg-Wohl, Sebastián Aveldaño, Sonia Salazar, Steve P Rogers, Sylke Frahnert, Thomas Valqui, Till Topfer, Tom Lawrence, Tom Matthews, Tony Parker, Victor Leandro, Vivien Chua, Yingqiang Lou, and Yoshika Willis.

All silhouettes shown in Figure 2 are unmodified, beyond their addition to the phylogenetic figure and re-colouring from black to grey. Images are attributed and credited as follows:

- *Fulmarus glacialis*, by Bennet McComish (photo by Avenue), CC BY-SA 3.0, http://phylopic.org/image/e8c26a02-7902-4abe-9b95-918efce22ac7/

- *Accipiter* (arbitrarily assigned to *Accipiter badius*), by Shyamal, CC BY 3.0, http://phylopic.org/image/f52d11cf-1d3c-41ae-b37e-e46c822a71b3/
- *Scopus umbretta*, by Arthur Grosset (photo), John E. McCormack, Michael G. Harvey, Brant C. Faircloth, Nicholas G. Crawford, Travis C. Glenn, Robb T. Brumfield & T. Michael Keesey, CC BY 3.0, http://phylopic.org/image/d0508bc3-dec1-40ee-85b1-a09b11a097b5/
- *Hirundo rustica*, by Matt Wilkins, CC0 1.0, http://phylopic.org/image/cf38be70-f0a8-4a47-b387-e797ec16c7c1/
- *Asio flammeus*, by Alexandre Vong, CC BY 3.0, http://phylopic.org/image/920f6a07-a4c6-4040-9c2e-9857481bbd6a/
- *Hirundapus caudacutus*, by Aviceda (vectorised by T. Michael Keesey), CC BY-SA 3.0, http://phylopic.org/image/690eb8a1-96f3-4b13-8f7d-544f8020abf8/
- *Oceanites oceanicus*, by Mark Hannaford (photo), John E. McCormack, Michael G. Harvey, Brant C. Faircloth, Nicholas G. Crawford, Travis C. Glenn, Robb T. Brumfield & T. Michael Keesey, CC BY 3.0, http://phylopic.org/image/15664833-6a30-408b-92e6-03329cce058e/
- *Cyanopsitta spixii*, by Sharon Wegner-Larsen, CC0 1.0, http://phylopic.org/image/34d9872c-b7d0-416f-8ac6-1f9f952982c8/
- *Trochilidae* (arbitrarily assigned to *Trochilus polytmus*), by Ferran Sayol, CC0 1.0, http://phylopic.org/image/2bf1e800-5384-45cd-a533-ac940b8eadd6/
- *Pyrrhocorax graculus*, by L. Shyamal, CC BY-NC-SA 3.0, http://phylopic.org/image/28951ec2-1ef3-437c-b1dc-4d54010732f7/
- *Apteryx* (arbitrarily assigned to *Apteryx australis*), by Ferran Sayol, CC0 1.0, http://phylopic.org/image/875bfb7c-99de-4140-bf6b-ce979c8bff06/
- *Columba livia*, by Dori <dori@merr.info> (source photo) and Nevit Dilmen, CC BY-SA 3.0, http://phylopic.org/image/966eed5e-b869-41b4-8de7-32286ae3656a/
- *Dendrocopos major*, by Steven Traver, CC0 1.0, http://phylopic.org/image/ddd5783c-ded5-48f2-a07d-cc37c83b227b/
- *Menura* (arbitrarily assigned to *Menura novaehollandiae*), uncredited, CC0 1.0, http://phylopic.org/image/217b650d-edaa-4f21-ac5d-03908fd5eac4/
- *Anas platyrhynchos*, by Sharon Wegner-Larsen, CC0 1.0, http://phylopic.org/image/d20848dc-7587-4a4e-8abd-3cc69c7a0f23/
- *Pitta guajana*, by Prin Pattawaro (photo), John E. McCormack, Michael G. Harvey, Brant C. Faircloth, Nicholas G. Crawford, Travis C. Glenn, Robb T. Brumfield & T. Michael Keesey, CC BY 3.0, http://phylopic.org/image/c3abc8bf-6d6c-4592-9f2d-5f7f75dca259/
- *Sayornis phoebe*, by Emily Willoughby, CC BY-SA 3.0, http://phylopic.org/image/ee6da2e5-95c1-431c-876f-9c6d7650ee97/
- *Zosterops* (arbitrarily assigned to Zosterops meeki), by Ferran Sayol, CC0 1.0, http://phylopic.org/image/9d382526-f8be-457a-b97d-f323a146b42a/
- *Emberiza bruniceps*, by L. Shyamal, CC BY-SA 3.0, http://phylopic.org/image/fb0ae249-66cc-4fce-b668-22ffabcb6bdc/
- *Passer domesticus,* by Andrew Butko, CC BY-SA 3.0, http://phylopic.org/image/3b74c3e5-1ffa-4089-95b1-371f1b71fce0/
- *Quiscalus quiscalus*, by Ferran Sayol, CC0 1.0, http://phylopic.org/image/2ada4e2f-35ab-48d2-b32e-3bad57033dd4/
- *Prunella modularis*, by Matt Wilkins, CC0 1.0, http://phylopic.org/image/88aeb901-6979-40a9-8991-b33dd8ac0a38/
- *Cistothorus platensis*, by Matt Wilkins, CC0 1.0, http://phylopic.org/image/adf1549b-e142-4a7a-bc9a-20d38ce7e1c8/
- *Vanellus coronatus*, by Rebecca Groom, CC BY 3.0, http://phylopic.org/image/8a5c8cde-0cd7-4ed6-876d-2415da7c00d6/

- *Athene noctua*, by Ferran Sayol, CC0 1.0, http://phylopic.org/image/4e35207e-a75a-4518-8eca-1d574eebfbb4/
- *Corvus brachyrhynchos*, by Peileppe, CC0 1.0, http://phylopic.org/image/2db31c7c-b0a9-460f-807e-da9181f21cf6/
- *Gallus gallus*, by Steven Traver, CC0 1.0, http://phylopic.org/image/2de1c95c-7e1f-429b-9c08-17f0a27d176f/
- *Gallirallus australis*, by T. Michael Keesey (vectorization) and HuttyMcphoo (photography), CC BY-SA 3.0, http://phylopic.org/image/d5194798-0b50-4a79-8b3d-e6efe468c5ab/
- *Phaenicophaeus curvirostris*, by Lip Kee Yap, CC BY-SA 3.0, http://phylopic.org/image/e2adf5e2-e7a8-4104-af3a-09afffe49cf3/
- *Pelecanus occidentalis*, by annaleeblysse, CC0 1.0, http://phylopic.org/image/8bbf9dc4-b846-4757-9d93-191a3e37b16f/

The CC BY-SA 3.0 license can be found at https://creativecommons.org/licenses/by-sa/3.0/ and the CC BY 3.0 license at https://creativecommons.org/licenses/by/3.0/. Information on CC0 1.0 (no copyright) can be found at https://creativecommons.org/publicdomain/zero/1.0/.

Supplementary References

1 Bates, D., Mächler, M., Bolker, B. & Walker, S. Fitting linear mixed-effects models using lme4. *J. Stat. Softw.* **67**, 1-48 (2015).
2 Barton, K. MuMIn: Multi-Model Inference. *R Foundation for Statistical Computing* (2019).
3 Pigot, A. L. *et al.* Macroevolutionary convergence connects morphological form to ecological function in birds. *Nat. Ecol. Evol.* **4**, 230-239 (2020).
4 Tobias, J. A. *et al.* Territoriality, social bonds, and the evolution of communal signaling in birds. *Front. Ecol. Evol.* **4**, 74 (2016).
5 Eyres, A., Böhning-Gaese, K. & Fritz, S. A. Quantification of climatic niches in birds: adding the temporal dimension. *J. Avian Biol.* **48**, 1517-1531 (2017).
