# Supplementary material for: Ecological drivers of global gradients in avian dispersal inferred from wing morphology
Source: Nat Commun. 2020 May 18;11:2463. doi: 10.1038/s41467-020-16313-6 (PMC7235233; doi:10.1038/s41467-020-16313-6)
Supplement: Supplementary file 2 — Reporting Summary [file 41467_2020_16313_MOESM2_ESM.pdf]

nature research

Corresponding author(s): Catherine Sheard
Last updated by author(s): Apr 6, 2020

Reporting Summary

Nature Research wishes to improve the reproducibility of the work that we publish. This form provides structure for consistency and transparency in reporting. For further information on Nature Research policies, see Authors & Referees and the Editorial Policy Checklist .

Statistics

For all statistical analyses, confirm that the following items are present in the figure legend, table legend, main text, or Methods section.

n/a	Confirmed	
☐	☒	The exact sample size (*n*) for each experimental group/condition, given as a discrete number and unit of measurement
☐	☒	A statement on whether measurements were taken from distinct samples or whether the same sample was measured repeatedly
☐	☒	The statistical test(s) used AND whether they are one- or two-sided<br>*Only common tests should be described solely by name; describe more complex techniques in the Methods section.*
☐	☒	A description of all covariates tested
☐	☒	A description of any assumptions or corrections, such as tests of normality and adjustment for multiple comparisons
☐	☒	A full description of the statistical parameters including central tendency (e.g. means) or other basic estimates (e.g. regression coefficient) AND variation (e.g. standard deviation) or associated estimates of uncertainty (e.g. confidence intervals)
☐	☒	For null hypothesis testing, the test statistic (e.g. *F*, *t*, *r*) with confidence intervals, effect sizes, degrees of freedom and *P* value noted<br>*Give P values as exact values whenever suitable.*
☐	☒	For Bayesian analysis, information on the choice of priors and Markov chain Monte Carlo settings
☐	☒	For hierarchical and complex designs, identification of the appropriate level for tests and full reporting of outcomes
☐	☒	Estimates of effect sizes (e.g. Cohen's *d*, Pearson's *r*), indicating how they were calculated

Our web collection on statistics for biologists contains articles on many of the points above.

Software and code

Policy information about availability of computer code

Data collection	No code was used to collect data.
Data analysis	R version 3.6.0, with packages MCMCglmm 2.29, sf 0.7-4, lme4 4_1.1-20, and MuMIn 1.43.14.

For manuscripts utilizing custom algorithms or software that are central to the research but not yet described in published literature, software must be made available to editors/reviewers. We strongly encourage code deposition in a community repository (e.g. GitHub). See the Nature Research guidelines for submitting code & software for further information.

Data

Policy information about availability of data

All manuscripts must include a data availability statement. This statement should provide the following information, where applicable:
- Accession codes, unique identifiers, or web links for publicly available datasets
- A list of figures that have associated raw data
- A description of any restrictions on data availability

The data supporting the results in this paper have been deposited at doi.org/10.5281/zenodo.3747657

Field-specific reporting

Please select the one below that is the best fit for your research. If you are not sure, read the appropriate sections before making your selection.

☐ Life sciences ☐ Behavioural & social sciences ☒ Ecological, evolutionary & environmental sciences

For a reference copy of the document with all sections, see nature.com/documents/nr-reporting-summary-flat.pdf

Ecological, evolutionary & environmental sciences study design

All studies must disclose on these points even when the disclosure is negative.

Study description	An investigation of the global drivers of wing morphological variation (a proxy for dispersal) in birds, from a new database of wing morphology measurements and compiled sources of ecological and environmental variables, using phylogenetic comparative methods.
Research sample	Wing morphological measurements for all extant and recently-extinct species for which we were able to find museum specimens or capture live birds (n=10,338 species, >99% of all extant birds). Measurements were taken from adult individuals, where possible in at least 2 males and 2 females, to capture a representative species average. Range and migration data is publicly available from birdlife.org; climate data from worldclim.org; phylogenetic data from birdtree.org; and territoriality, habitat use, and additional migratory data from Tobias et al. 2016.
Sampling strategy	Morphological measurements were obtained for all available bird species. An average of 4.43 adult individuals were measured per species to provide reliable mean species values.
Data collection	Morphological data were obtained using callipers and wing rulers from live caught individuals and preserved museum skins, by the nine authors and the 90 collaborators and assistants thanked in the Supplementary Notes. Ecological and social data were obtained from the literature, and biogeographic and climate data from BirdLife International, WorldClim, and other sources, by CS and JAT.
Timing and spatial scale	Spatial scale: Global.<br>Timing: Data is based on museum collections spanning the last ~200 years and on recent fieldwork. There was no systematic sampling based on museum specimen age, other than selecting specimens in good condition to better preserve the collections. Most measurements were taken 2011-2015, with some as early as the mid-1990s and as late as 2018.
Data exclusions	Specimens labelled or identified in-hand as juvenile were excluded, as we were interested in intraspecific variation in adult morphology/behaviour and not in ontogeny. This exclusion was pre-determined.
Reproducibility	Inter-measurer repeatability was measured by taking replicate measurements for 220 specimens for 146 species; measurer identity explained 0.01-0.5% of the variation in measured traits. Differences in measurements taken on museum specimens and live birds explained 0.002-0.35% of the variation in measured traits, across 4,018 specimens in 362 species. Analyses run on two alternative sets of migration scores produced minimal differences in results.
Randomization	NA
Blinding	NA

Did the study involve field work? ☐ Yes ☒ No

Reporting for specific materials, systems and methods

We require information from authors about some types of materials, experimental systems and methods used in many studies. Here, indicate whether each material, system or method listed is relevant to your study. If you are not sure if a list item applies to your research, read the appropriate section before selecting a response.

Materials & experimental systems

n/a	Involved in the study	
☒	☐	Antibodies
☒	☐	Eukaryotic cell lines
☒	☐	Palaeontology
☐	☒	Animals and other organisms
☒	☐	Human research participants
☒	☐	Clinical data

Methods

n/a	Involved in the study	
☒	☐	ChIP-seq
☒	☐	Flow cytometry
☒	☐	MRI-based neuroimaging

Animals and other organisms

Policy information about studies involving animals; ARRIVE guidelines recommended for reporting animal research

Laboratory animals	No laboratory animals were used
Wild animals	Most data are from museum specimens. Some data are included from wild-caught birds that were not harmed during data collection and subsequently released into the wild. In all cases, birds were caught by mist-netting, a passive, non-invasive technique which does not harm the individual birds.
Field-collected samples	No samples were taken from the field

Ethics oversight	Natural Environment Research Council

Note that full information on the approval of the study protocol must also be provided in the manuscript.
